# Aluminum Stress Response Is Regulated Through a miR156/SPL13 Module in *Medicago sativa*

**DOI:** 10.3390/genes16070751

**Published:** 2025-06-27

**Authors:** Gamalat Allam, Solihu K. Sakariyahu, Binghui Shan, Banyar Aung, Tim McDowell, Yousef Papadopoulos, Mark A. Bernards, Abdelali Hannoufa

**Affiliations:** 1 Agriculture and Agri-Food Canada, 1391 Sandford Street, London, ON N5V 4T3, Canada; gamalat.allam@agr.gc.ca (G.A.); solihukayode.sakariyahu@agr.gc.ca (S.K.S.); binghui.shan@agr.gc.ca (B.S.); tim.mcdowell@agr.gc.ca (T.M.); 2 Department of Biology, University of Western Ontario, 1151 Richmond Street, London, ON N6A 3K7, Canada; banyae.ong@gmail.com (B.A.); bernards@uwo.ca (M.A.B.); 3 Agriculture and Agri-Food Canada, 58 River Road, Truro, NS B2N 5E3, Canada; yousef.papadopoulos@agr.gc.ca

**Keywords:** Al toxicity, *Medicago sativa*, miR156, SPL13, transcriptome profiling, ChIP-seq, root development

## Abstract

Background: Aluminum (Al) toxicity severely limits *Medicago sativa* (alfalfa) production on acidic soils, resulting in major yield losses worldwide. The highly conserved miRNA156 (miR156) functions by downregulating at least 11 SQUAMOSA promoter-binding protein-like (SPL) transcription factors in alfalfa, including SPL13, but its role in Al stress remains unclear. This study aimed to investigate the miR156/SPL regulatory network’s function in alfalfa under Al stress. Methods: Gene expression analyses, histochemical staining, nutrient profiling, phenotypic assays, transcriptome profiling, and ChIP-seq were conducted on alfalfa plants with altered miR156 and SPL13 expression to assess their roles in the Al stress response. Results: Al stress induced SPL13 expression while repressing miR156 in the roots. Elevated miR156 intensified Al accumulation, lipid peroxidation, and plasma membrane damage, accompanied by reduced leaf nitrogen, magnesium, sulfur, and phosphorus content. Phenotypically, increased SPL13 enhanced the root length and Al tolerance, whereas SPL13 silencing reduced tolerance. Transcriptome profiling of SPL13-silenced plants identified differentially expressed genes involved in the Al response, including aluminum-activated malate transporters and various transcription factors (GRAS, Myb-related, bHLH041, NAC, WRKY53, bZIP, and MADS-box). ChIP-seq revealed that SPL13 directly regulates genes encoding a protein kinase, cytochrome P450, and fasciclin-like arabinogalactan proteins. Conclusions: The MsmiR156/MsSPL13 network plays a crucial regulatory role in alfalfa’s response to Al toxicity. These findings provide novel genetic targets and foundational knowledge to advance molecular breeding for enhanced Al tolerance in alfalfa.

## 1. Introduction

Al toxicity is a critical factor limiting crop growth and productivity in acidic soils; it is known to affect nearly 50% of global arable land [1], contributing to food shortages. Al^3+^ is the most toxic form of Al, inhibiting root elongation at micromolar concentrations in a short period of time [2]. Al stress cause severe root damage in *M. truncatula* [3].

Al toxicity significantly impairs nutrient uptake in plants by interfering with the absorption, transport, and utilization of essential nutrients, such as calcium (Ca), magnesium (Mg), phosphorus (P), and potassium (K) [4]. Al binds with soil phosphorus, causing P deficiency, inducing oxidative stress, and disrupting root respiration and the functioning of the cell wall and plasma membrane [5]. This toxicity impedes root cell division, growth, and ion transport mechanisms, such as H^+^-ATPase activity [6]. Studies have shown that Al toxicity reduces Ca and Mg uptake in maize and wheat [7,8]; Ca, K, Cu, Co, Mg, Zn, Mo, Mn, Ni, and S levels in pea [9]; and N, P, K, Ca, Mg, and S in citrus [10]. Al decreases the nutrient efficiency and causes P deficiency by forming insoluble compounds in sugarcane [11]. These disruptions lead to nutritional imbalances, impairing crop growth and development, including in alfalfa under highly acidic conditions [12,13,14]. Plants have evolved mechanisms to withstand Al toxicity. For example, the enhanced expression of polygalacturonase1 (*MsPG*1) in *M. sativa* root apical epidermal cells reduced Al accumulation in the cell wall, thereby enhancing Al tolerance [15]. Plants are also known to secrete organic acids such as malic acid, citric acid, and oxalic acids to chelate Al^3+^ and form Al–organic acid complexes, thereby reducing Al transport to root cells and the harmful effects of Al [16].

Alfalfa (*M. sativa*) is a globally significant legume forage crop, valued for its exceptional palatability and high protein content [17]. Beyond its primary role in animal nutrition, alfalfa is recognized as a promising low-input bioenergy crop due to its substantial yield potential and energy efficiency [18,19]. In 2024, the global alfalfa hay market reached an estimated volume of approximately 285 million metric tons [20], underscoring the crop’s importance and the pressing need to develop high-performing cultivars. However, conventional breeding methods in alfalfa face significant challenges arising from its autotetraploid genome (2n = 4x = 32) and inherent self-incompatibility [21]. Genetic engineering has emerged as a powerful alternative, rapidly generating transgenic plants with enhanced traits, including improved yields and increased stress tolerance [22]. Recent advances in alfalfa genomics, particularly through full-length transcript sequencing and chromosome-level genome assemblies, offer a robust foundation for molecular breeding strategies [23,24,25,26].

microRNA156 (miR156) is a conserved small RNA in plants that modulates plant growth and development by silencing SQUAMOSA promoter-binding protein-like (*SPL*) genes [27]. SPLs are transcription factors with a conserved 76-amino-acid SBP domain containing a zinc-finger structure and a nuclear localization signal, enabling them to bind the “TNCGTACAA” consensus sequence and regulate downstream genes [28,29]. SPL proteins influence several plant development processes, including the root architecture [30,31], stem elongation [22,32], flowering time [22,32], seed weight [33], and branching [34]. The overexpression of miR156 affects agronomic traits and stress responses across various plants by targeting SPLs [27]. For instance, overexpressing miR156a in tomato increased inflorescences and branches but reduced the flower and fruit yields by suppressing *SPL13* [35]. In Arabidopsis, the miR156-mediated silencing of *SPL9* improved the tolerance to salinity and osmotic stress and enhanced anthocyanin accumulation [36], while enhanced miR156 expression levels decreased the apical bud size and shortened the primary roots, with SPL10 silencing affecting cytokinin responses, thereby reducing apical bud activity and shoot regeneration [31]. Likewise, in wheat, tae-miR156 overexpression increased tiller numbers by repressing SPLs [37]. Conversely, in apples, miR156 overexpression reduced salt resistance, whereas MdSPL13 enhanced it [38]. While a role for miR156 in response to aluminum stress has been proposed [39,40,41,42], there have been no studies on the specific SPLs involved in this type of stress.

At the molecular level, a defense strategy against Al involves the regulatory role of microRNAs (miRNAs) in certain transcription factors (TFs) [43]. These include GRAS TF family members, known for their vital roles in responding to both biotic and abiotic stresses and their influence on root and meristem development [44]. In *Medicago truncatula*, GRAS TF genes showed notable upregulation during the early stages of Al exposure [43]. Similarly, the MYB TF family, essential in regulating cell wall biosynthesis and stress responses [45], is modulated by various differentially expressed (DE) miRNAs under Al stress conditions [43]. The WRKY family, characterized as plant-specific zinc-finger TFs, participates in complex signaling networks and stress regulation [46,47]. The expression of WRKY TFs is likewise modulated by miRNAs under Al stress [43]. Additionally, the basic helix–loop–helix (bHLH) TF family, among the largest TF families in plants, regulates critical pathways involved in light signaling, hormonal responses, and root hair development, thus contributing significantly to stress tolerance [48]. Notably, in *M. truncatula*, 10 differentially expressed miRNAs coordinately upregulate the GRAS, MYB, WRKY, and bHLH TF families at the onset of Al stress. This coordinated regulation highlights the essential role of miRNAs in regulating transcriptional responses that support early root growth and ultimately enhance Al resistance [43].

In previous work, we showed that MsmiR156 negatively regulated alfalfa’s response to Al stress [49]. The current study further elucidates the regulatory mechanisms underlying the MsmiR156/MsSPL13 pathway in alfalfa’s response to Al stress. Focusing specifically on MsmiR156d, we investigated how the MsmiR156/MsSPL13 regulatory network influences downstream genes that enhance Al tolerance in alfalfa. Our experimental approach included histochemical analyses to assess the impact of MsmiR156 overexpression and Al toxicity on plant growth, coupled with nutrient profiling to evaluate how a reduction in root length affects nutrient uptake. Transcript-level analyses were performed to determine the expression patterns of the *MsmiR156* and *MsSPL* genes under Al stress. Phenotypic assessments of plants with either overexpressed or silenced SPL13 further clarified its specific role in root growth regulation under Al stress. Moreover, transcriptomic analyses of *SPL13*-RNAi alfalfa plants identified the differential expression of genes in response to Al stress, while chromatin immunoprecipitation sequencing (ChIP-seq) revealed target genes directly regulated by SPL13. This study represents the first comprehensive characterization of the regulatory function of the MsmiR156/MsSPL13 network in modulating Al stress tolerance in alfalfa.

## 2. Materials and Methods

### 2.1. Plant Materials and Growth Conditions

Transgenic alfalfa (*M. sativa*) plants expressing miR156 (miR156-OE) genotypes (designated A11a, A17, and A8) were previously generated and characterized at Agriculture and Agri-Food Canada, as described in [22]. WT plant clone N4.4.2 [50] was obtained from Dr. Daniel Brown (Agriculture and Agri-Food Canada). Previously characterized transgenic alfalfa plants with altered *SPL13* expression, including *SPL13*-RNAi genotypes (SPL13-RNAi02, SPL13-RNAi05, and SPL13-RNAi06) [51], and 35S::*SPL13-GFP* [52] were used in this study. The transgenic and WT alfalfa plants were grown in a fully automated greenhouse with 16 h light (380–450 W/m^2^), 70% relative humidity (RH), and a constant temperature of 25 ± 2 °C at the Agriculture and Agri-Food Canada London Research and Development Center, London, ON, Canada. Due to alfalfa’s propensity for outcrossing, all plants used in this study were propagated through vegetative cuttings to maintain the genotype’s consistency. The vegetative propagation and morphological characterization of alfalfa plants were carried out using the method described in [22].

### 2.2. Generation of 35S:SPL13-OE Constructs and Plant Transformation

To investigate the regulatory role of SPL13 in alfalfa under Al stress, SPL13 overexpression genotypes (SPL13-OE1, SPL13-OE61, and SPL13-OE304) were generated, harboring the full-length coding sequence of SPL13 (1135 bp). The SPL13 sequence was first amplified from alfalfa cDNA using primers OEMsSPL13-F and OEMsSPL13-R (Table S1) and cloned into the pENTR/D-TOPO entry vector (Invitrogen, Carlsbad, CA, USA). PCR screening and Sanger sequencing confirmed the successful cloning of the insert. The SPL13 fragment was then recombined into the pMDC32 gateway destination vector [53] using LR Clonase (Invitrogen, Carlsbad, CA, USA), with the reaction incubated overnight at room temperature. The resulting construct was transformed into *E. coli*-competent cells using heat shock [54], and successful recombinants were confirmed by Sanger sequencing. The *SPL13* overexpression construct was transformed into *Agrobacterium tumefaciens* (LBA4404 or EHA105) using heat shock [55]. Transformed *A. tumefaciens* strains were used to transform alfalfa N.4.4.2 germplasm through a tissue culture-based method [22]. Transgenic *SPL13*-OE alfalfa plants were validated using a 35S promoter (35S-F3)- and gene-specific reverse primer (OEMsSPL13-R), while control plants containing the empty vector were confirmed using a combination of the 35S promoter and pMDC32 vector-specific primers (Table S1). Transcript levels of the *SPL13* gene were analyzed through qRT-PCR using gene-specific primers Ms-SPL13Fq1-F and Ms-SPL13Rq1 (Table S1).

### 2.3. Al Treatment

To investigate the regulatory role of miR156/SPL13 in the alfalfa response to Al stress, MsmiR156-OE, MsSPL13-OE, MsSPL13-RNAi, and WT plants were subjected to Al stress. Rooted stem cuttings of these genotypes were initially transferred to 5″ plastic pots, each filled with an equal amount of soil mix, and allowed to grow for 35 days. The plants were then transferred to 3.2 L plastic buckets containing a half-strength aerated nutrient solution [56] with an adjusted pH of 4.5 for a 14-day acclimatization period. Following acclimatization, the 49-day-old plants were treated with either 100 μM AlCl_3_·6H_2_O solution or no Al (0 μM Al) for 14 days. Fresh AlCl_3_·6H_2_O was applied every three days to maintain consistent Al exposure throughout the treatment period.

### 2.4. Histochemical Staining

Histochemical staining was used to examine the effects of miR156 on Al accumulation and the impact on plant functions. To assess Al accumulation, alfalfa plants were exposed to two Al treatments, 0 µM and 100 µM AlCl_3_, for 30 min, 4 h, 8 h, and 24 h in a hydroponic culture, as described in Section 2.3. After treatment, the longest roots of the 49-day-old alfalfa plants were thoroughly rinsed in distilled water for 1 h and then immersed in a hematoxylin solution (0.2% *w*/*v* hematoxylin in 0.02% *w*/*v* KIO_3_) for 10 min, as described [57]. Subsequently, the roots were rinsed with repeated changes of distilled water for 1 h and assessed for the degree of hematoxylin staining in the root tips. The staining patterns of the root tips were captured using a stereomicroscope (Nikon, SMZ 1500, Tokyo, Japan).

To evaluate plasma membrane injury due to lipid peroxidation, root tips were submerged in Schiff’s reagent for 10 min and then rinsed with 0.5% potassium metabisulfite (K_2_S_2_O_5_; *w*/*v*) in 0.05 M HCl, as described in Tistama, Widyastuti, Sopandie, Yokota, and Akashi [57]. The stained roots were kept in sulfite solution to retain the staining color. These stained roots were observed under the abovementioned stereomicroscope (Nikon, SMZ 1500, Tokyo, Japan).

To assess and localize the loss of plasma membrane integrity, the roots were immersed in 10 mL of Evans Blue solution [0.025% (*w*/*v*) Evans Blue in 100 mM CaCl_2_, pH 5.6] for 10 min, as described in Tistama, Widyastuti, Sopandie, Yokota, and Akashi [57]. The stained roots were rinsed three times with 200 mL of 100 mM CaCl_2_ (pH 5.6) and root tips were subsequently analyzed under a stereomicroscope (Nikon, SMZ 1500, Tokyo, Japan).

### 2.5. Nutrient Analysis

To determine the effects of miR*156* and Al stress on the levels of essential nutrients, a nutrient analysis of WT and miRNA156-OE plants exposed to Al was conducted as a fee for service by A&L Canada Laboratories, London, ON, Canada. Samples consisting of approximately 5 g of alfalfa leaf tissue were collected and then dried in an oven at 60 °C for five days until a constant weight was achieved. The dried tissue was then ground into a 2 mm powder using a mechanical grinder. For analysis, 0.5 g of each sample was weighed and digested with 5 mL of concentrated nitric acid (HNO_3_), first at room temperature for 30 min (pre-digestion) and then heated to 120–150 °C until fully digested. To ensure complete digestion, 1–2 mL of hydrogen peroxide (H_2_O_2_) was added, followed by further heating when necessary. The digested samples were diluted with deionized water to a final volume of 50 mL. Nitrogen content was analyzed using inductively coupled plasma optical emission spectroscopy (ICP-OES) according to EPA methods 3050B and 6010B, with the same solution used for magnesium, sulfur, and phosphorus analyses. The concentrations of N, Mg, S, and P, were expressed as a percentage or parts per million (ppm) of dry weight.

### 2.6. RNA Extraction and Sequencing

Fresh leaf samples from alfalfa plants, both Al-treated (100 μM) and untreated (0 μM), were collected, immediately flash-frozen in liquid nitrogen, and stored at −80 °C until required for subsequent RT-qPCR analysis and RNA sequencing. For total RNA extraction, approximately 100 mg of leaf tissue was processed using the QIAGEN RNeasy^®^ Plant Mini Kit (Qiagen, Germantown, MD, USA, Cat # 74904), following the manufacturer’s protocol. Homogenization was conducted with a PowerLyzer^®^24 bench-top bead-based homogenizer (MO BIO Laboratories, Inc., Carlsbad, CA, USA, Cat # 13155), following the manufacturer’s instructions. The integrity and concentration of the total RNA extracted were confirmed using both a BioRad Bioanalyzer and Nanodrop prior to proceeding with RNA sequencing. High-quality RNA samples were then submitted to Novogene (Sacramento, CA, USA) for mRNA-stranded library preparation and sequenced for 150-bp paired-end reads on an Illumina NovaSeq platform (Illumina, Inc., San Diego, CA, USA), generating a minimum of 60 million reads per sample as a fee for service.

### 2.7. RNA-Seq Analysis

The sequenced paired-end raw reads (FASTQ format) were processed and analyzed using the Digital Research Alliance of Canada High-Performance Computing (HPC) resources (https://ccdb.alliancecan.ca/, accessed on 15 September 2023) with custom Linux shell scripts (provided in Table S2). Briefly, the quality control of the raw reads was assessed using FastQC [58], and low-quality reads, along with Illumina adapter sequences, were removed using Trimmomatic v0.39 [59] with the following settings: ILLUMINACLIP:TruSeq3-PE.fa:2:30:10LEADING:3 TRAILING:3 SLIDINGWINDOW:4:15 MINLEN:36. The clean reads were then aligned to the *M. sativa* (alfalfa) reference genome [24], downloaded from https://figshare.com/articles/dataset/genome_fasta_sequence_and_annotation_files/12327602 (accessed on 20 September 2023) using Hisat2 v2.2.1 [60], with the --dta flag enabled to allow downstream transcriptome assembly, and -k 1 was used to report only uniquely mapped reads. The resulting sorted BAM alignment files were used for transcript quantification using featureCounts [61], enabling the accurate assessment of the transcript abundance for downstream analysis.

### 2.8. Differential Gene Expression, Gene Ontology, and GTAC Motif Analyses

To investigate the transcriptional response to Al stress, RNA-seq data analysis was performed on WT and SPL13-RNAi genotypes treated with 100 µM Al or Al 0 µM Al (control), using three biological replicates per treatment. Differentially expressed genes (DEGs) were identified using DESeq2 [62], with significance defined by a threshold of log2 (fold change) ≥ 0 and adjusted *p*-value ≤ 0.05. To assess variance and sample clustering, principal component analysis (PCA) was conducted using the prcomp function in R. Shared and unique DEGs between conditions were visualized through stacked bar plots and upset plots, following a script adapted from Bjornson et al. [63]. Scaled expression profiles of Al-stressed and control plants were visualized in a heatmap using “geom_tile” in ggplot2 [64], offering a detailed overview of transcriptomic changes.

A Gene Ontology (GO) enrichment analysis of the DEGs was conducted using the topGO package in R [65] to identify biological processes, molecular functions, and cellular components associated with the DEGs. GO terms were considered significantly enriched for DEGs at *p* < 0.05 using the Fisher test. The enrichment of the core GTAC SPL binding elements on the promoters of the DEGs was performed using SEA [66], while the individual motif occurrence on the 2 kb promoter of a DEG was assessed using FIMO [67] of the MEME suite [68]. The sequence logo representation of GTAC-enriched motifs in DEG promoter regions was visualized using WebLogo (https://weblogo.berkeley.edu/, accessed on 12 March 2024) [69].

### 2.9. Validation of RNA-Seq Results by RT-qPCR

The RNA-seq results were validated by RT-qPCR using 11 randomly selected differentially expressed genes and an aliquot of the same RNA used for RNA-seq. For this, extracted RNA was treated with Ambion^®^ TURBO DNA-free™ DNase (Invitrogen, Waltham, MA, USA, Cat # AM1907), followed by iScript™ cDNA synthesis (Invitrogen, Waltham, MA, USA, Cat # 1708891). Specific gene targets were selected for validation as listed in Supplementary Table S3. Primers for these genes were designed using the Primer3 software (http://primer3.ut.ee/, accessed on 20 April 2024) based on the *M. sativa* genome sequence, and amplified products were sequenced for verification. Primer efficiency was checked before proceeding with RT-qPCR analysis. RT-qPCR was performed using the CFX96™ Real-Time PCR Detection System (Bio-Rad, Hercules, CA, USA) and SsoFast™ EvaGreen^®^ Supermix (Bio-Rad, Hercules, CA, USA, Cat # 1725204). Each 10 μL reaction contained 2 μL cDNA (equivalent to 200 ng), 1 μL of each forward and reverse primer (10 μM), 5 μL SsoFast EvaGreen Supermix(Bio-Rad, Hercules, CA, USA, Cat # 1725204), and 2 μL nuclease-free water. The PCR amplification conditions were initial denaturation at 95 °C for 30 s, followed by 40 cycles of 95 °C for 10 s, 58 °C for 30 s, and 72 °C for 30 s, with a final melting curve analysis from 65 °C to 95 °C, incrementing by 0.5 °C every 5 s. All reactions included three technical replicates. Data analysis was performed with the CFX Maestro™ software (version 3.1) (Bio-Rad). Transcript levels were normalized against the housekeeping genes acetyl-CoA carboxylase 1 (ACC1) and acetyl-CoA carboxylase 2 (ACC1), with primers designed from the alfalfa sequence [70], and relative gene expression ratios were calculated using the Vandesompele method [71].

### 2.10. Identification of Transcription Factors (TFs) Under Al Stress

To explore the transcriptional response to Al stress, transcription factors (TFs) expressed in *M. sativa* were identified using the previously published alfalfa TF database [72]. TFs with differential expression under Al stress were then visualized in a heatmap, providing insights into their regulatory dynamics and potential roles in the Al tolerance mechanism.

### 2.11. Identification and SBP Domain Analysis of SPL13 Candidates

A local BLASTX analysis was performed using the SPL13 nucleotide sequence from previous studies against the predicted protein database derived from the *M. sativa* genome annotation [24]. The BLASTX (version 2.11.0) search was conducted with default parameters, and candidate genes were selected based on stringent criteria: an e-value threshold of <1 × 10^−200^ and minimum sequence identity of 97%. The protein sequences corresponding to the candidate *SPL13* genes were retrieved from the *M. sativa* proteome dataset. To validate the presence of the conserved SBP domain (PF03110) within these candidate proteins, an HMM-based profile search was conducted using HMMER (hmmsearch), with an e-value threshold set at 0.001. Candidates positively identified as containing the SBP domain were retained for further characterization.

### 2.12. Analysis of the SPL13 Coexpression Network Under Al Stress

A coexpression-based gene regulatory network was inferred using GENIE3 [73]. The target genes used in network construction included DEGs and the entire set of expressed transcription factors. The potential regulatory relationships between target genes and the transcription factors were identified through FIMO by scanning the 1 kb promoters of the target genes for the binding motif of each TF. The results from FIMO were structured into gene-specific customized TF–target gene lists for input into GENIE3. Genes lacking significant motifs or associated TF matches were excluded. The inferred network was filtered to include interactions with weights > 0.01, balancing network complexity between sparse and excessively dense topologies. The inferred coexpression network was visualized using Cytoscape (version3.10.0) [74] via the RCy3 R package (version 3.21).

### 2.13. Small RNA Target Analysis in Alfalfa

To determine whether miR156 targets the three copies of SPL13 in *M. sativa*, a plant small RNA target prediction analysis was performed. The mature miR156 sequence, along with the full sequences of the three MsSPL13 copies, was input into the psRNATarget online tool, namely psRNATarget: A Plant Small RNA Target Analysis Server (2017 Update) (zhaolab.org). This tool predicted potential miR156 binding sites within the *SPL13* gene sequences. The results were subsequently analyzed to explore the regulatory role of miR156 in the expression of SPL13, specifically examining whether miR156 modulates SPL13 expression through mechanisms such as mRNA cleavage or translation inhibition (Table S4 in Supplementary Materials).

### 2.14. Chromatin Immunoprecipitation (ChIP) Assay

To determine genomic sequences that were occupied by SPL13, a ChIP-seq analysis was conducted using leaf tissue from one-month-old p35S:SPL13-GFP and WT alfalfa plants under control conditions, as previously described [75], with minor modifications. Briefly, approximately 5 g of fresh leaf tissue from p35S:SPL13-GFP and WT plants was collected, washed, and cross-linked with 1% formaldehyde for 20 min under a vacuum. The cross-linked tissue was ground into a fine powder using liquid nitrogen. Extraction buffers and reagents are listed in Table S5. The fine powder tissue was homogenized in a 5 mL tube using pre-chilled buffer S and incubated on a rotator at 4 °C for 30 min. The homogenate was then divided into five aliquots of 600 μL. The chromatin was sheared into fragments of 100–500 bp using a sonicator (Sonic Dismembrator, Fisher Scientific, Pittsburgh, PA, USA) with three 15 s pulses each at 50% amplitude and a 20 s pause. Following sonication, the chromatin was centrifuged at 14,000× *g* for 10 min. A 20 μL aliquot of the supernatant was kept at −20 °C as an input DNA control, and the remainder was subjected to immunoprecipitation. The chromatin solution was divided into two equal parts and diluted 4 times using pre-chilled buffer F in a 15 mL tube. The diluted solution was pre-incubated with pre-washed protein A agarose beads (Millipore, Oakville, ON, Canada) on a rotator at 4 °C for 1 h. Each sample received 30 μL of beads, followed by a magnetic frame to collect the beads. The supernatant was transferred to a new tube, and 2 μL of Ab290 GFP antibody (Abcam, Cambridge, UK, Cat# ab290) was added to one part of the chromatin solution for immunoprecipitation. The antibody–bead complex was incubated overnight at 4 °C with gentle rotation. The immune complexes were recovered by the magnetic frame and subjected to sequential washes with buffers of varying ionic strength (low salt, high salt, LiCl, and TE buffer). The immunocomplexes were eluted from the beads with 250 μL of elution buffer two times, and cross-linking was reversed by incubating with 20 μL of 5 M NaCl at 65 °C overnight, including the input control. The samples were treated with 10 μL of 0.5 M EDTA, 20 μL of 1 M Tris-HCl (pH 6.5), and 2 μL of 10 mg/mL proteinase K (Sigma-Aldrich, Oakville, ON, Canada). The DNA was purified using the MinElute PCR Purification Kit (Qiagen, Toronto, ON, Canada, Cat# 28004), following the manufacturer’s instructions. Finally, the quantity and quality of the recovered DNA were assessed using a Qubit fluorometer (Thermo Fisher Scientific, Pittsburgh, PA, USA) to ensure suitability for subsequent sequencing and analysis.

### 2.15. ChIP-Seq and Data Analyses

The ChIP-seq libraries were constructed using a minimum of 2 ng of each ChIP DNA sample, with each sample represented by two biological replicates. High-throughput sequencing of these libraries was carried out on the Illumina NovaSeq platform as a fee for service at Novogene (Sacramento, CA, USA). The resulting sequence data were analyzed following established protocols [75]. Initially, the raw data were mapped to the *M. sativa* genome [24] using Bowtie2 with the default settings [76,77], retaining only uniquely mapped reads for subsequent analyses. The read counts for all ChIP-seq experiments and the pipeline of ChIP-seq analysis are listed in the Supplementary Materials (Tables S6 and S7). Peak identification, indicating regions of read enrichment, was performed using MACS2.0 [78] with the following parameters: “gsize = 1,864,511,547, bw = 300, q = 0.05, nomodel, ext size = 200”. Integrative Genomics Viewer (IGV) [79] was employed for data visualization. Only peaks that were present in both biological replicates were considered for further analysis. ChIPseeker was used with default settings to annotate the peaks to genes. Enriched DNA motifs at SPL13-bound sites were identified using the DREME/MEME suite, focusing on 300 bp sequences surrounding each peak summit (150 bp upstream and downstream) (http://meme-suite.org/tools/meme-chip, accessed on 18 April 2024) [80]. computeMatrix, plotHeatmap, and plotProfile were used to compare the mean occupancy density.

### 2.16. Statistical Analysis

Statistical analyses were performed using R (version 4.2.3). A one-way ANOVA was used to evaluate the effects of 100 µM Al stress on miR156 and SPL gene expression at 0, 8, and 24 h in WT plants, comparing miR156 and SPL13 expression at each time point, as well as SPL13 expression in SPL13 overexpression plants under control conditions. To assess the impact of miR156 overexpression on the nutrient levels (N, Mg, S, P) in the miR156-OE and WT genotypes under both 100 µM Al stress and control (0 µM Al) conditions, two-way ANOVAs were conducted. Additionally, two-way ANOVAs were used to examine Al stress’ effects on the root morphology across the MsSPL13-OE, SPL13-RNAi, and WT genotypes compared to controls. A post hoc Tukey’s multiple-comparison test was applied to all significant results, with the significance threshold set at *p* ≤ 0.05. Groups sharing the same letter were considered statistically non-significant. The statistical analysis of the RNA-seq data was conducted using R (version 4.4.1) [81]. The Linux shell and R scripts used to analyze and visualize the RNA sequence data are available in Table S2.

## 3. Results

### 3.1. miR156 Exacerbates Al Toxicity

**Figure 1:**
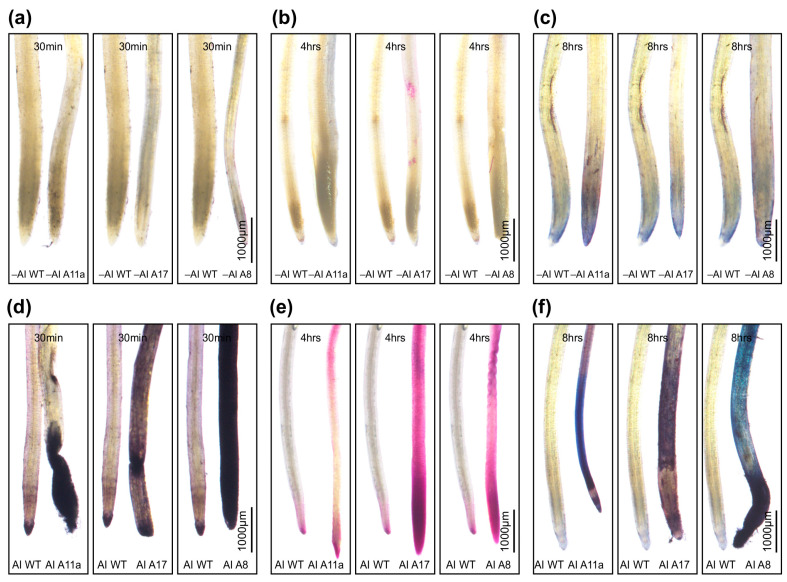
Histochemical staining of roots of MsmiR156 overexpression (miR156-OE) plants (A11a, A17, and A8) and WT treated with 100 µM Al (Al) and untreated (-Al) control. No-Al controls ((**a**–**c**) panels); 100 uM Al (**d**–**f**) treatment. Aluminum accumulation in the root tips of WT and miR156-OE genotypes detected by staining with hematoxylin for 30 min (**a**,**d**). Lipid peroxidation in the root tips of WT and miR156-OE genotypes detected by staining with Schiff’s reagent for 4 h (**b**,**e**). Loss of plasma membrane detected by staining with Evan’s Blue for 8 h (**c**,**f**). Scale bar = 100 µm.

To investigate the effects of MsmiR156 on the severity of Al stress in alfalfa, MsmiR156-OE (A11a, A17, and A8) and WT plants were exposed to either 100 µM Al or no Al (0 µM) for 30 min, 4 h, and 8 h in a hydroponic culture. The histochemical staining of alfalfa roots with hematoxylin revealed that plants with enhanced expression of MsmiR156 accumulated Al more intensively after 30 min (Figure 1a,d). Staining with Schiff’s reagent revealed lipid peroxidation after 4 h (Figure 1b,e), and staining with Evan’s Blue showed a loss of plasma membrane integrity after 8 h (Figure 1c,f) relative to WT under Al stress conditions. These findings suggest that increasing the expression of MsmiR156 enhances the severity of Al toxicity in alfalfa.

### 3.2. miR156 Affects Nutrient Uptake Under Al Stress

**Figure 2:**
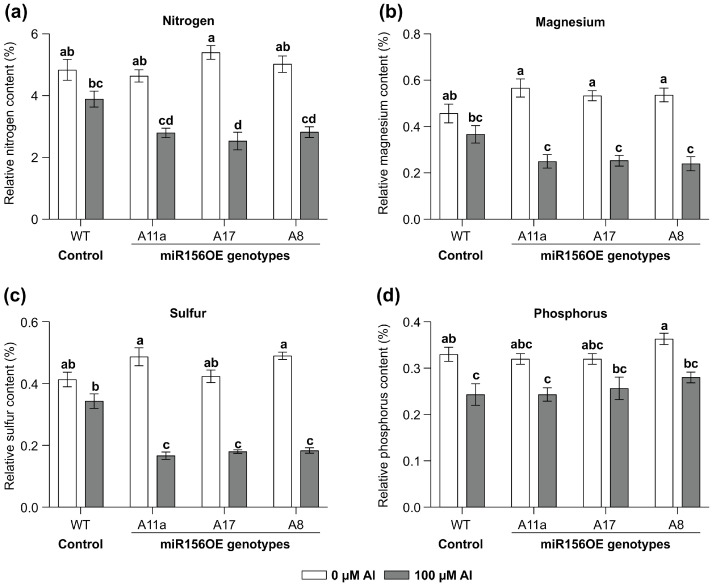
Effects of miR156 overexpression on nutrient levels in alfalfa leaves grown under Al stress. Nitrogen (**a**); magnesium (**b**); sulfur (**c**); and phosphorus (**d**). Each bar plot represents the mean. Error bars represent SEM. Two-way ANOVA was conducted with n = 3 individual plants. Significant differences detected from the two-way ANOVA in R (version R-4.2.3), followed by a post hoc Tukey multiple-comparison test. Means with the same letter are not significantly different at *p* < 0.05.

To explore the impact of MsmiR156-OE on roots and nutrient uptake under Al stress, MsmiR156-OE and WT alfalfa plants were exposed to 100 uM Al for 14 days in a hydroponic culture and the leaf N, Mg, S, and P content assessed (Figure 2). Nutrient uptake in miR156-OE plants was significantly restricted (Figure 2). While no differences in nitrogen content could be observed between WT and miR156-OE under no Al treatment, there was a reduction of 53% in A17, 44% in A11a, and 40% in A8 under Al conditions compared to untreated controls (Figure 2a). Although WT plants also exhibited reduced nitrogen levels under Al, the reduction was less pronounced than in miR156-OE plants (Figure 2a). Additionally, while there was no significant difference in magnesium content between miR156-OE and WT plants under no Al, MsmiR156-OE plants showed substantial magnesium reductions of 55% in A17 and A11a and 53% in A8 but only 19% in WT compared to their untreated counterparts (Figure 2b). Furthermore, the sulfur content was significantly lower in all miR156-OE genotypes compared to WT under Al stress. When comparing the same genotypes treated with Al stress to their untreated counterparts, the sulfur reductions were 66% in A11a, 63% in A17, and 57% in A8, compared to 17% in WT (Figure 2c). For phosphorus, all alfalfa plants showed a decrease, with WT and A8 plants showing the most significant reductions relative to their untreated counterparts (Figure 2d). These findings suggest that miR156-OE negatively impacts nutrient uptake in alfalfa under Al stress.

### 3.3. MsmiR156 Regulates MsSPL13 in Response to Al

**Figure 3:**
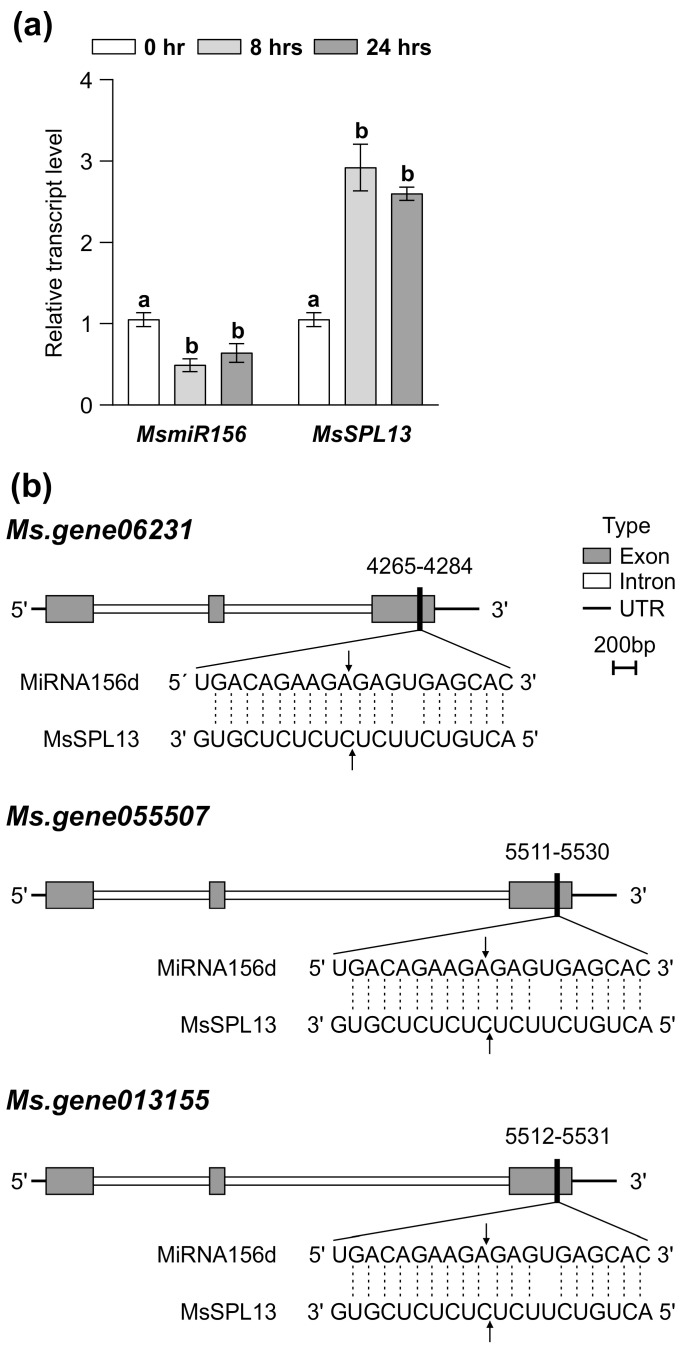
Effects of Al stress on the expression of MsmiR156 and MsSPL13 genes in WT alfalfa roots. Transcript levels of MsmiR156 and MsSPL13 (**a**) after 0, 8, and 24 h of Al treatment and the binding site of miR156 on the three different copies of the MsSPL13 gene (**b**). Each bar plot represents the mean. Error bars represent (SEM). A one-way ANOVA was conducted with n = 4 individual plants. Significant differences detected from the one-way ANOVA in R (version R-4.2.3), followed by a post hoc Tukey multiple-comparison test. Means with the same letters are not significantly different at *p* ≤ 0.05.

MsmiR156 targets a network of at least 11 *SPL* genes in alfalfa for transcript cleavage, including *MsSPL2a*, *MsSPL3*, *MsSPL4*, *MsSPL6*, *MsSPL7a*, *MsSPL8*, *MsSPL9*, *MsSPL11*, *MsSPL12*, *MsSPL13*, and *MsSPL13a* [22,82,83,84]. To identify which *MsSPL* genes were targeted by MsmiR156 under Al stress, WT alfalfa plants were exposed to treatment with 100 µM AlCl_3_ for 0, 8, and 24 h in a hydroponic culture. *MsmiR156d* expression decreased over time under Al stress (Figure 3a), while *MsSPL13* expression increased during the same period (Figure 3a).

The expression patterns of the other 10 *MsSPL* genes were also analyzed under Al stress (Figure S1a–i). These genes exhibited three distinct expression patterns over 24 h: *MsSPL13a*, *MsSPL2a*, *MsSPL7a*, *MsSPL9*, and *MsSPL4* showed an initial increase followed by a decrease; *MsSPL8* decreased and then increased; and *MsSPL3* consistently decreased (Figure S1a–i). Notably, none of these patterns matched the clear inverse expression correlation of *SPL13* and *MsmiR156*, highlighting their regulatory relationship.

Previously, a 5′-RACE analysis confirmed that *SPL13* was targeted by *miR156*, with cleavage sites within its binding regions identified in alfalfa [22]. To further investigate whether *miR156* targets the three copies of *SPL13* and to explore its regulatory role in *SPL13* expression, we conducted a plant small RNA target (PSRNA target) analysis. Using the mature sequence of *MsmiR156* and the full sequences of the three copies of *MsSPL13* (*MS. gene013155*, *MS. gene055507*, and *MS. gene06231*), our analysis revealed an expectation value of 1 for all interactions, indicating a high confidence level that miR156d targets these genes. The results demonstrated that miR156d facilitates the mRNA degradation of these targets, with cleavage occurring at specific positions: 5521 in *MS. gene013155*, 5520 in *MS. gene055507*, and 4274 in *MS. gene06231*, all occurring within the third exon (Figure 3b and Table S4 in Supplementary Materials). These cleavage sites in the middle of the miRNA–mRNA alignment correspond to typical locations for RNA-induced silencing complex (RISC) activity in plants [85,86], demonstrating the precision of *MsmiR156*-mediated regulation. Collectively, these findings highlight *MsmiR156* as an essential regulator of *MsSPL13* under Al stress in alfalfa.

### 3.4. SPL13 Regulates Root Growth Under Al Stress

**Figure 4:**
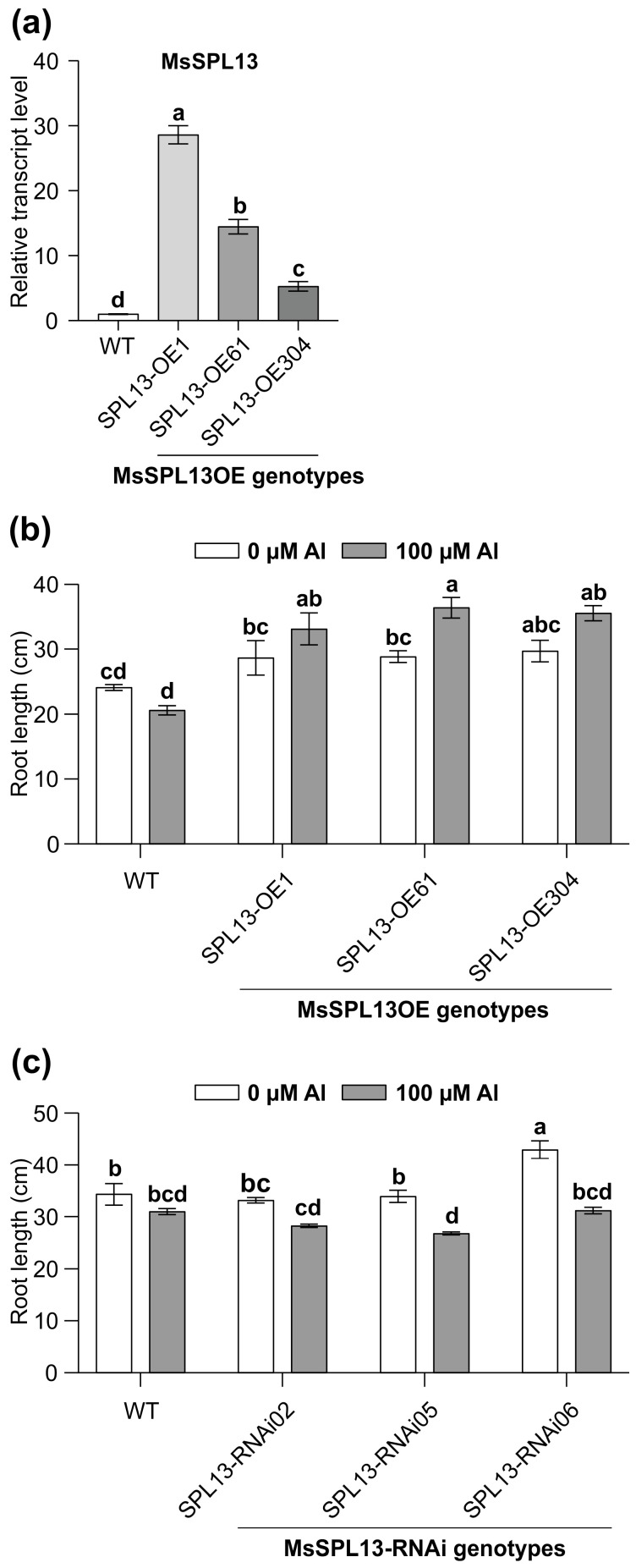
Effects of Al treatment on root length in MsSPL13-OE and MsSPL13-RNAi plants. (**a**) Expression profile of MsSPL13 in leaves of one-month-old MsSPL13-OE alfalfa plants. In panel (**a**), white bars indicate expression levels in WT, light grey bars represent the highest *MsSPL13* expression in SPL13-OE1, medium grey indicates moderate expression in SPL13-OE61, and dark grey indicates the lowest expression in SPL13-OE304, (**b**) root length in WT and SPL13-OE plants, (**c**) root length in WT and MsSPL13-RNAi plants. Each bar plot represents the mean. Error bars represent (SEM). Statistical analyses were conducted using one-way ANOVA for MsSPL13 expression data (n = 5 individual plants) and two-way ANOVA for root length (n = 6 individual plants for MsSPL13-OE; n = 9–10 individual plants for MsSPL13-RNAi). Significant differences detected from the one-way ANOVA and two-way ANOVA in R (version R-4.2.3), followed by a post hoc Tukey multiple-comparison test. Means with the same letters are not significantly different at *p* ≤ 0.05.

The inhibition of root growth is one of the main impacts of Al on plants [12]. To explore the influence of MsSPL13 on the roots under Al stress, the *SPL13*-overexpressing (SPL13-OE1, SPL13-OE61, SPL13-OE304), *SPL13*-silenced (SPL13-RNAi02, SPL13-RNAi05, SPL13-RNAi06), and WT plants were exposed to 100 µM Al for 14 days in a hydroponic culture. As expected, the transcript levels of *SPL13* were higher in SPL13-OE genotypes relative to WT under no-Al control conditions, with the highest in SPL13-OE1, moderate in SPL13-OE61, and low in SPL13-OE304 (Figure 4a). Phenotypic analysis under both Al-stressed and control conditions revealed contrasting responses among SPL13-OE and SPL13-RNAi plants compared to WT (Figure 4b,c). All SPL13-OE plants showed an increase in root length under Al stress, with SPL13-OE61 showing a particularly notable increase in root length compared to its untreated counterpart (Figure 4b). On the other hand, SPL13-RNAi plants did not show significant changes in root length under Al stress; however, SPL13-RNAi05 and SPL13-RNAi06, with moderate to high silencing of SPL13 [87], revealed a substantial reduction in root length relative to their untreated controls (Figure 4c). These results underscore the critical function of SPL13 in modulating root growth in response to Al stress in alfalfa.

### 3.5. Global Changes in Gene Expression in MsSPL13-RNAi Plants Under Al Stress

**Figure 5:**
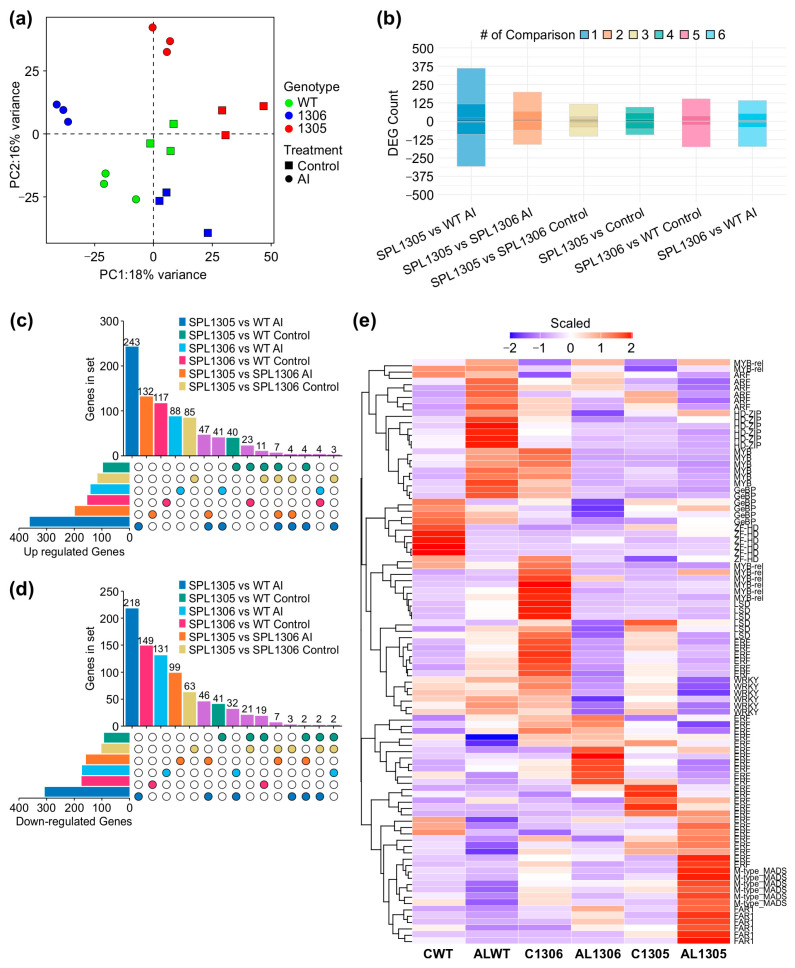
Comparison of differentially expressed genes (DEGs) between WT and SPL13-RNAi genotypes (SPL13-RNAi05 and SPL13-RNAi06) under Al stress (100 µM Al) relative to no-Al control (0 µM Al). Control groups are designated as CWT (WT), C1305 (SPL13-RNAi05), and C1306 (SPL13-RNAi06), while Al-treated groups are represented as Al WT, Al 1305 (SPL13-RNAi05), and Al 1306 (SPL13-RNAi06). PCA plot (**a**), stacked bar plot of differentially expressed genes under Al compared to no-Al control (**b**), UpSet plot of upregulated genes (**c**), in panels (**c**,**d**), purple bars represent genes shared across multiple comparisons, UpSet plot of downregulated genes (**d**), heatmap of differentially expressed transcription factor genes (**e**).

To uncover *MsmiR156/MsSPL13*-regulated genes that are potentially involved in Al stress responses, high-throughput RNA-seq analysis was performed on leaf tissue from *MsSPL13*-RNAi (SPL13-RNAi05, SPL13-RNAi06) and WT plants grown under Al and control conditions (Supplementary Materials). The sequencing yielded 1,182,411,630 raw reads and 1,162,654,940 clean reads, achieving 98.33% of clean base coverage. Each sample generated between 72,405,401 and 64,255,127 clean reads, averaging 64,591,941.11 per sample, with a mapping rate to the *M. sativa* reference genome [24] of 91.39% and 93.24%, respectively (Table S8). Under control conditions, the average read count was approximately 65,709,202.17 for *SPL13*-RNAi and 61,731,091.33 for WT, whereas, under Al stress, the average read counts were about 63,409,195.83 and 67,583,759.33 in *SPL13*-RNAi and WT, respectively (Table S8). These read counts were used to conduct a differential gene expression analysis. Under control conditions, the average number of reads mapped to genes was approximately 42,705,843.67 for *SPL13*-RNAi and 39,934,507 for WT. Under Al stress, the average number of reads mapped to genes was about 42,024,130.67 for *SPL13*-RNAi and 44,445,305 for WT (Table S8). Differential gene expression analysis followed by principal component analysis (PCA) revealed the distinct clustering of samples by genotype and treatment, explaining 18% of the variance despite minor outliers (Figure 5a). This clustering indicates clear transcriptional differences between the groups, reflecting a genotype-specific response to Al stress. It is worth noting that some variability among biological replicates may be expected due to minor differences in microclimate or developmental stage. Overall, this finding shows clear separation among groups of samples. We then assessed the differential gene expression between *SPL13*-RNAi and WT plants under both control and Al stress conditions (Table S9 in Supplementary Materials). A total of 6149 differentially expressed genes (DEGs) were identified, including 2909 upregulated and 3240 downregulated genes, in *SPL13*-RNAi and WT plants under both Al stress and control conditions (Table S9). Of these, 964 genes were upregulated and 912 downregulated. Specifically, 329 upregulated and 332 downregulated genes were unique to the control conditions, whereas 636 upregulated and 580 downregulated genes were specific to Al stress (Table S9, Figure 5b–d). This indicates that the transcriptional differences between *SPL13*-RNAi and WT were more pronounced under Al stress than under control conditions. Interestingly, a subset of 117 DEGs was commonly regulated in both *SPL13*-RNAi plants (SPL13-RNAi05 and SPL13-RNAi06). Among these, 41 genes were upregulated and 32 downregulated under Al stress, while 21 genes were downregulated and 23 upregulated under control conditions (Figure 5b–d, Table S9). Key downregulated genes potentially directly regulated by *SPL13* included protein kinases, cytochrome P450, and fasciclin-like arabinogalactan proteins (FLAs). Protein kinases and cytochrome P450 are known for their role in the Al toxicity response [88,89,90]. Interestingly, two aluminum-activated malate transporters showed the opposite expression patterns—one upregulated and the other downregulated. Members of the heavy metal-associated domain superfamily, critical for Al detoxification, were downregulated, possibly as indirect effects of *SPL13* regulation. Conversely, genes such as small auxin-up RNA (SAUR) and auxin-induced proteins were upregulated, potentially contributing to adaptive responses to Al stress [91] and likely regulated indirectly by *SPL13*. These findings shed light on the transcriptional networks governed by *MsmiR156/MsSPL13* under Al stress, emphasizing key genes and pathways that enhance alfalfa’s adaptive mechanisms.

### 3.6. Differential Expression of Transcription Factors in MsSPL13-RNAi Alfalfa Under Al Stress

Transcription factors (TFs) play a central role in plant responses to Al stress. Prior studies in *Medicago truncatula* have identified key TF families, including GRAS, MYB, WRKY, and bHLH, as differentially expressed in response to Al toxicity [43]. To explore this response in alfalfa, we conducted a comparative transcriptomic analysis to identify differentially expressed transcription factors (DETFs) between *MsSPL13*-RNAi and WT plants under Al stress and control conditions. Our analysis revealed that a total of 119 TFs showed differential expression in *MsSPL13*-RNAi compared to WT under Al stress (Figure 5e and Table S10 in Supplementary Materials). Among these, 70 TFs were downregulated while 49 were upregulated in *MsSPL13*-RNAi. A more detailed examination of the 70 downregulated TFs showed that 48 were specifically suppressed under Al stress, whereas only 22 displayed reduced expression under control conditions (Figure 5e, Table S10). Conversely, of the 49 upregulated TFs, 34 were induced under Al stress, while 15 showed increased expression under control conditions (Figure 5e, Table S10). Further comparison between *MsSPL13*-RNAi genotypes (*SPL13-RNAi05* and *SPL13-RNAi06*) identified 18 commonly downregulated TFs under Al stress, compared to nine under control conditions, while 11 TFs were consistently upregulated under Al stress, with only two shared under control conditions (Figure 5e, Table S10). Among the upregulated TFs were the GRAS family, MYB, and bHLH041 (Figure 5e, Table S10), which play essential roles in the Al response in *Medicago tructula* [43], suggesting a conserved role in alfalfa. Additionally, *MADS-box TFs*, *NAC domain-containing* proteins, and bZIP transcription factors were upregulated under Al stress (Figure 5e, Table S10). MADS-box TFs and NAC domain-containing proteins are critical for Al tolerance [92,93], while bZIP TFs—essential regulators of plant responses to various abiotic stresses, including heavy metals [94]—likely play a central role in the Al stress response in alfalfa. In contrast, downregulated TFs included WRKY, WRKY53, MYB4-like, and C_2_H_2_-type zinc-finger proteins (ZFP) (Figure 5e, Table S10), all of which are known to be involved in Al stress responses [95,96]. These TFs, whether upregulated or downregulated, are likely indirectly regulated by *MsSPL13* and contribute to transcriptional changes under Al stress. Collectively, these findings shed light on critical molecular regulatory mechanisms that contribute to alfalfa’s adaptive responses to Al stress.

**Figure 6:**
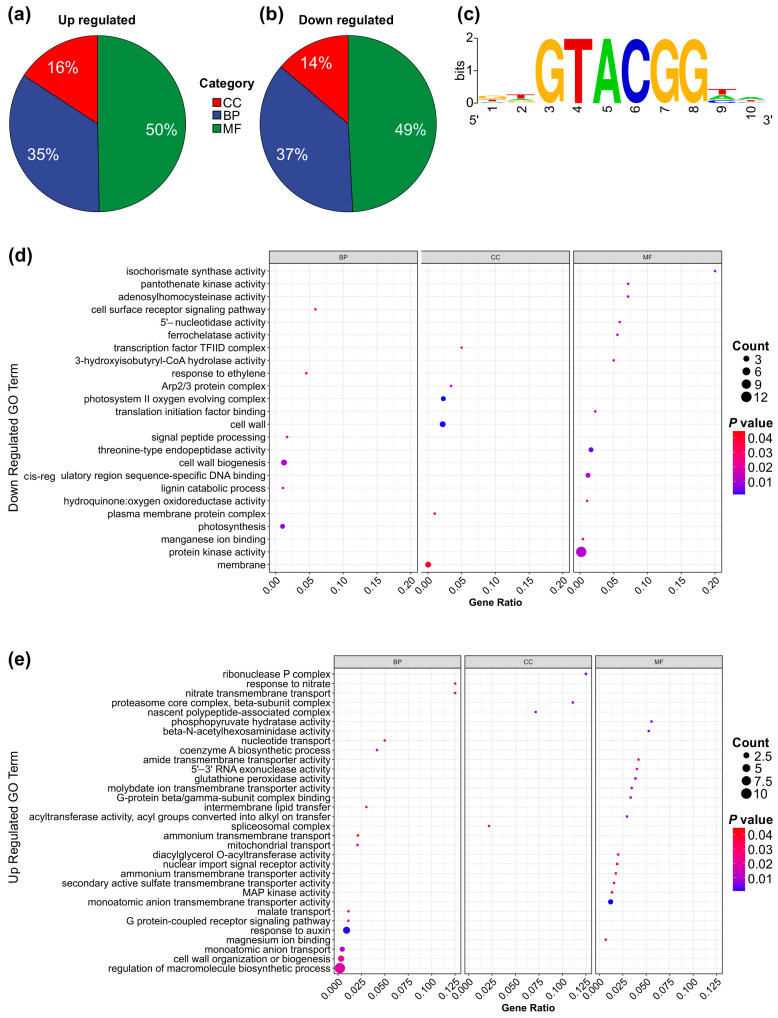
GO term enrichment and functional responses of differentially expressed genes (DEGs) in SPL13-RNAi compared to WT. Pie chart illustrating the distribution of upregulated DEGs across GO categories (**a**): cellular component (CC), biological process (BP), and molecular function (MF). Pie chart depicting the distribution of downregulated DEGs across the GO categories: CC, BP, and MF (**b**). Enrichment of core GTAC motif in the promoters of all DEGs (**c**). Dot plot representing the enriched GO terms for CC, BP, and MF among the upregulated DEGs (**d**). Dot plot showcasing the enriched GO terms for CC, BP, and MF among the downregulated DEGs (**e**).

A Gene Ontology (GO) enrichment analysis was conducted to investigate the functional roles of differentially expressed genes in SPL13-RNAi compared to WT under Al stress and control conditions. The analysis revealed that 16% of upregulated genes and 14% of downregulated genes were associated with cellular components (Figure 6a,b,d,e and Figure S2b,c and Table S11 in Supplementary Materials). Additionally, 35% of upregulated genes and 37% of downregulated genes were involved in biological processes (Figure 6a,b,d,e and Figure S2b,c, Table S11). Furthermore, 50% of upregulated and 49% of downregulated genes were linked to molecular functions (Figure 6a,b,d,e and Figure S2b,c, Table S11). The GO term enrichment analysis highlighted those upregulated genes involved in malate transport, cell wall organization or biogenesis, and the auxin response. In contrast, downregulated genes involved protein kinase activity, membrane transport, and photosynthesis (Figure 6d,e and Figure S2b,c, Table S11). Notably, the enriched motif in all differentially expressed genes was GGTACGG, with a GTAC core sequence (Figure 6c and Figure S2a), which is typical of miR156-regulated genes [97,98]. These results suggest significant functional differences in gene expression between SPL13-RNAi and WT under Al stress and control conditions.

### 3.7. Genotype-Specific Response of Alfalfa to Al Stress

**Figure 7:**
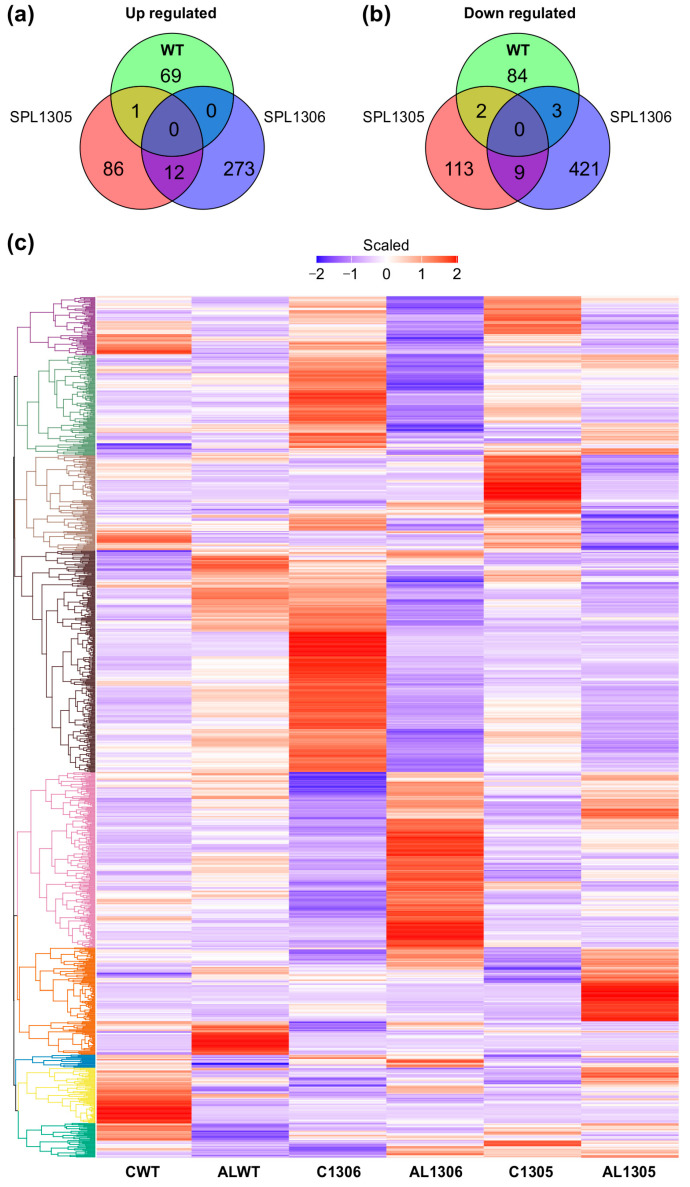
Genotype-specific comparison of DEGs in WT and SPL13-RNAi plants (SPL13-RNAi05 and SPL13-RNAi06) under Al stress (100 µM Al) relative to no-Al control (0 µM Al). Control groups are designated as CWT (WT), C1305 (SPL13-RNAi05), and C1306 (SPL13-RNAi06), while Al-treated groups are represented as Al WT, Al 1305 (SPL13-RNAi05), and Al 1306 (SPL13-RNAi06). Venn diagram illustrating the overlap and uniqueness of upregulated DEGs between WT and RNAi genotypes (**a**), Venn diagram depicting the overlap and uniqueness of downregulated DEGs between WT and RNAi genotypes (**b**), and heatmap displaying the expression patterns of all DEGs in WT and RNAi genotypes under both control and Al stress conditions (**c**).

To elucidate the transcriptomic impact of SPL13 silencing on alfalfa’s response to Al tolerance, changes in global gene expression in the leaf tissue of SPL13-RNAi-silenced and WT alfalfa plants were examined under Al stress. Compared to their untreated counterparts, Al-treated WT plants had 69 upregulated and 84 downregulated genes (Figure 7a–c and Table S12 in Supplementary Materials), whereas the SPL13-05 genotype had 86 upregulated and 113 downregulated genes (Figure 7a–c, Table S12) and the SPL13-06 genotype showed 273 upregulated and 421 downregulated genes compared to their untreated counterparts (Figure 7a,b, Table S12). An analysis of commonly differentially expressed genes revealed one upregulated and two downregulated genes shared between SPL13-05 and WT (Figure 7a–c, Table S12). No common upregulated genes were identified between SPL13-06 and WT, but there were three downregulated genes (Figure 7b,c, Table S12). There were 12 common upregulated and nine downregulated genes between SPL13-05 and SPL13-06 under Al stress (Figure 7a–c, Table S12). SPL13-RNAi genotypes exhibited more differentially expressed genes under Al stress than WT, indicating a more pronounced transcriptomic response to Al stress when SPL13 is silenced.

### 3.8. GO Analysis of Genotype-Specific Comparison of DEG Pathways Modulating Al Stress Response in MsSPL13-RNAi Alfalfa

**Figure 8:**
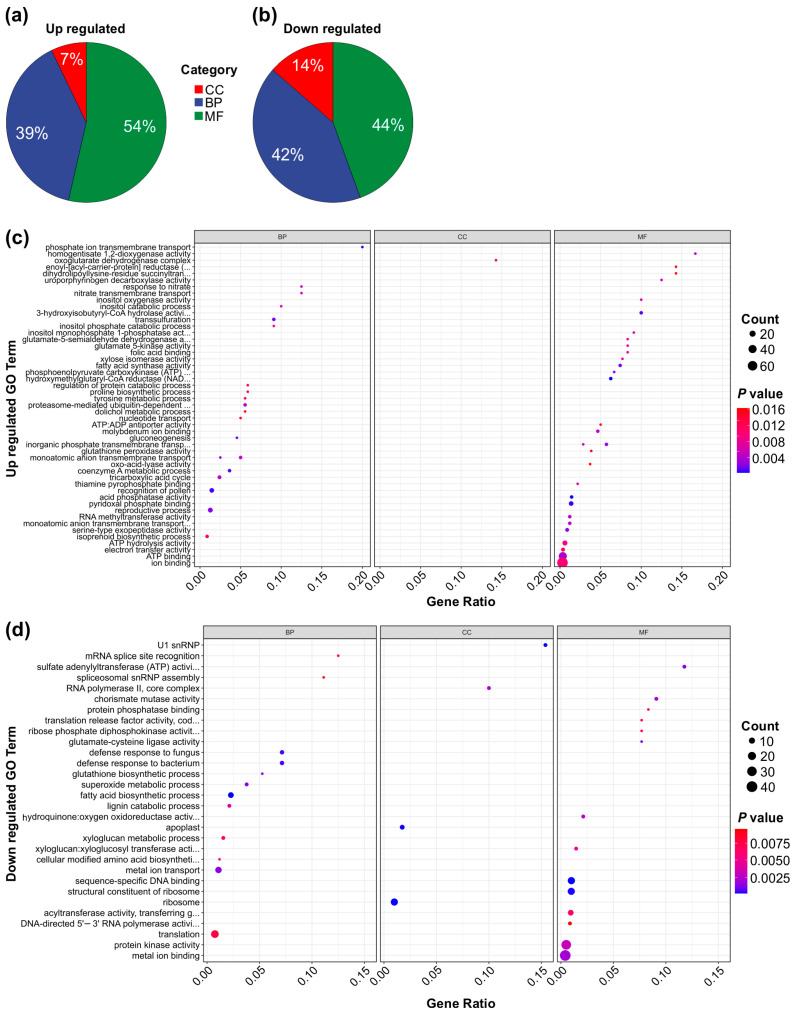
GO term analysis of genotype-specific transcription of DEGs in SPL13-RNAi and WT plants under Al stress and control conditions. Pie chart displaying the distribution of upregulated TFs across the GO categories: cellular component (CC), biological process (BP), and molecular function (MF) (**a**). Pie chart showing the distribution of downregulated TFs across the same GO categories: CC, BP, and MF (**b**). Dot plot depicting the enriched GO terms for upregulated genes (**c**). Dot plot depicting the enriched GO terms for downregulated genes (**d**).

To further elucidate the functional roles of the DEGs in the genotype-specific comparison, we performed a Gene Ontology (GO) term analysis comparing SPL13-RNAi and WT plants under both Al and control conditions. This analysis categorized DEG involvement across cellular components, biological processes, and molecular functions. Among the upregulated DEGs, 7% were associated with cellular components, 39% with biological processes, and 54% with molecular functions (Figure 8a,c and Figure S3a). Conversely, among the downregulated DEGs, 14% were associated with cellular components, 42% with biological processes, and 44% with molecular functions (Figure 8b,d and Figure S3b). Functionally, upregulated DEGs were significantly enriched in pathways related to ion binding, ATP binding, electron transfer activity, monatomic anion transmembrane transport, reproductive processes, the tricarboxylic acid (TCA) cycle, and nitrate transport (Figure 8a,c and Figure S3a). In contrast, downregulated DEGs showed involvement in protein kinase activity, metal ion transport, metal ion binding, xyloglucan metabolic processes, fatty acid biosynthesis, sequence-specific DNA binding, and translation (Figure 8b,d and Figure S3b). Together, these findings highlight the transcriptional changes induced by MsSPL13 silencing under Al stress, revealing key molecular pathways that contribute to alfalfa’s response to Al stress.

### 3.9. Regulatory Network and Functional Enrichment of SPL13-Associated Genes Under Al Stress

**Figure 9:**
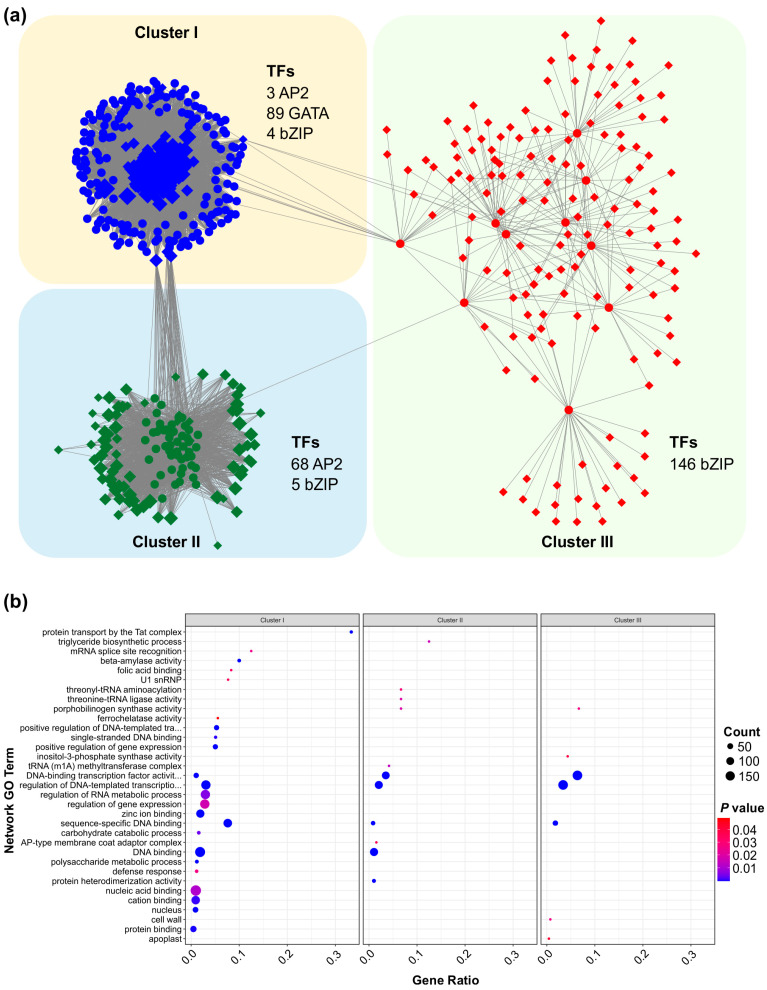
Analysis of the SPL13 gene regulatory network under Al stress. Network visualization (**a**) and GO term enrichment analysis of genes in network clusters (**b**).

To uncover the regulatory mechanisms of SPL13 under Al stress, we performed a comprehensive gene regulatory network analysis of the differentially expressed genes (DEGs) between SPL13-RNAi and WT plants. This analysis revealed three distinct clusters of transcription factors (TFs) and associated genes, each displaying distinct connectivity patterns and regulatory influences (Figure 9 and Table S13 in Supplementary Materials). Cluster I primarily comprised 89 GATA TFs and showed a highly coordinated expression pattern, with additional contributions from three AP2 and five bZIP TFs (Figure 9, Table S13). The dense connectivity within this cluster suggests a vital role for GATA TFs in mediating gene expression responses to Al stress. Cluster II predominantly consisted of 68 AP2 and five bZIP TFs and formed a moderately connected network, indicating a distinct regulatory module compared to Cluster I (Figure 9a, Table S13). In contrast, Cluster III, the largest group, comprised 146 bZIP TFs and their associated genes, indicating an extensive regulatory network that likely plays a crucial role in transcriptional modulation under Al stress (Figure 9a, Table S13). The interconnectivity between these clusters highlights the cooperative interplay of different TF families in modulating gene expression under Al stress.

Functional insights into these regulatory networks were further explored through Gene Ontology (GO) enrichment analysis (Figure 9b, Table S13). Cluster I genes exhibited significant enrichment in GO terms related to protein transport by the Tat complex, the triglyceride biosynthetic process, mRNA splice site recognition, and various enzymes’ activity, such as beta-amylase activity (Figure 9b, Table S13). These results suggest a complex response involving lipid metabolism, mRNA processing, and enzymatic regulation under Al stress. Cluster II was significantly enriched in threonyl-tRNA ligase activity, the positive regulation of gene expression, and single-stranded DNA binding (Figure 9b, Table S13), aligning with the well-established roles of AP2 TFs in transcriptional and translational regulation. Meanwhile, cluster III was associated with DNA-binding transcription factor activity, the regulation of DNA-templated transcription, and carbohydrate catabolic processes (Figure 9b, Table S13), emphasizing the essential role of bZIP TFs in transcriptional regulation and the metabolic response under stress conditions. Collectively, these findings reveal a complex and diverse regulatory network governed by SPL13 under Al stress, involving multiple TF families and a broad range of biological processes. This integrated regulatory mechanism highlights the dynamic interplay of TFs in modulating the gene expression of DEGs to respond to Al stress in alfalfa.

### 3.10. Genome-Wide Identification of Genes Regulated Directly by SPL13 in Alfalfa

**Figure 10:**
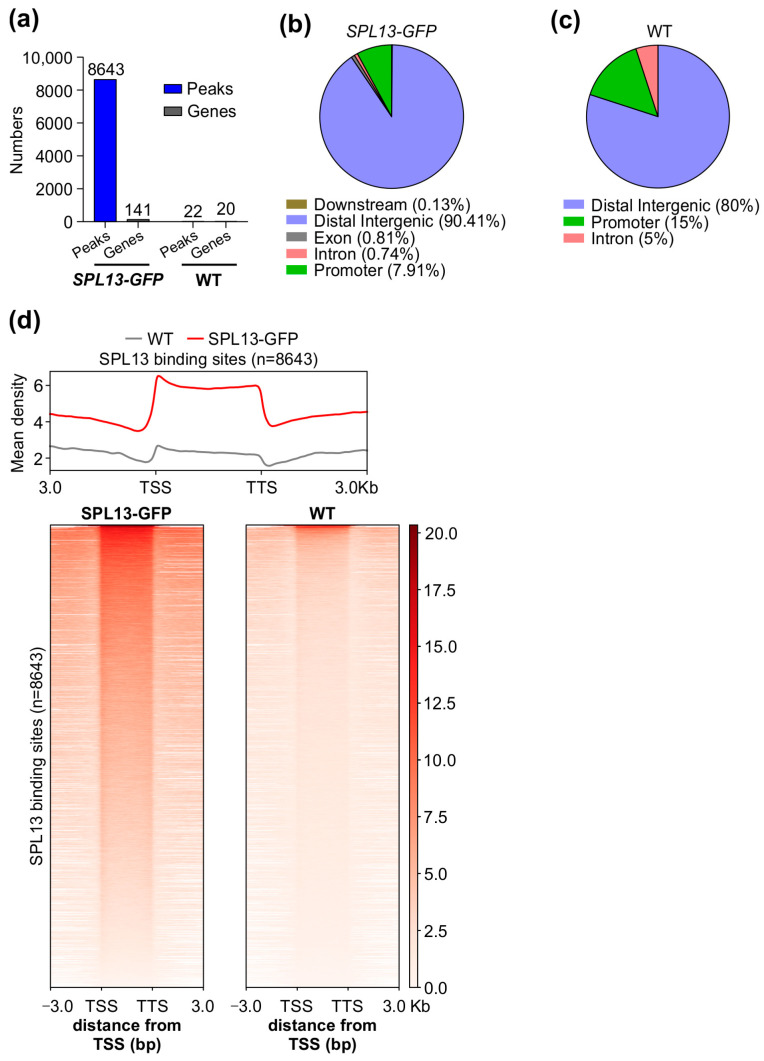
ChIP-seq analysis of SPL13-GFP and WT plants. Number of MsSPL13 binding sites (peaks) and genes in the SPL13-GFP plant and WT (**a**). Pie chart showing the distribution of SPL13 peaks in the downstream region, distal intergenic region, exon, intron, and promoter in the genome (**b**). Pie chart showing the distribution of WT peaks in the distal intergenic region, intron, and promoter in the genome (**c**). Metagene plot and heatmap representing the mean density of SPL13 binding sites and SPL13 occupancy at all sites in SPL13-GFP compared with WT (**d**).

To further investigate the SPL13 gene regulatory network, we conducted chromatin immunoprecipitation sequencing (ChIP-seq) on alfalfa plants expressing 35S::*SPL13-GFP*, comparing them to WT under control conditions. The analysis revealed a markedly higher number of SPL13 binding sites in the genomes of 35S::*SPL13-GFP* plants than in WT (Figure 10a and Tables S14 and S15 in the Supplementary Materials), with SPL13-GFP having 8643 peaks associated with 141 genes, relative to WT having only 22 peaks corresponding to 20 genes (Figure 10a, Tables S14 and S15). This indicates that SPL13 overexpression leads to increased binding site occupancy, suggesting that the higher availability of SPL13 results in more binding events. The identified binding sites were distributed across various genomic regions, including promoters, exons, introns, distal intergenic regions, and downstream sequences, with notable enrichment in promoter regions. Additionally, substantial occupancy was observed in distal intergenic and downstream regions (Figure 10b,c and Table S16 in Supplementary Materials). These distribution patterns suggest that SPL13 primarily regulates gene expression through interaction with promoter regions, while also influencing gene activity via distal and downstream elements. Further analysis of the SPL13 binding density using heatmaps and metagene plots confirmed the significant enrichment near transcription start sites (TSS), supporting SPL13’s critical role in gene activation and transcriptional regulation (Figure 10d). The increased SPL13 occupancy, particularly at the promoter regions, aligns with the distribution in the pie chart (Figure 10b,c, Table S16), highlighting SPL13’s broad regulatory function. These findings indicate that SPL13 directly binds to DNA to regulate gene expression networks.

**Figure 11:**
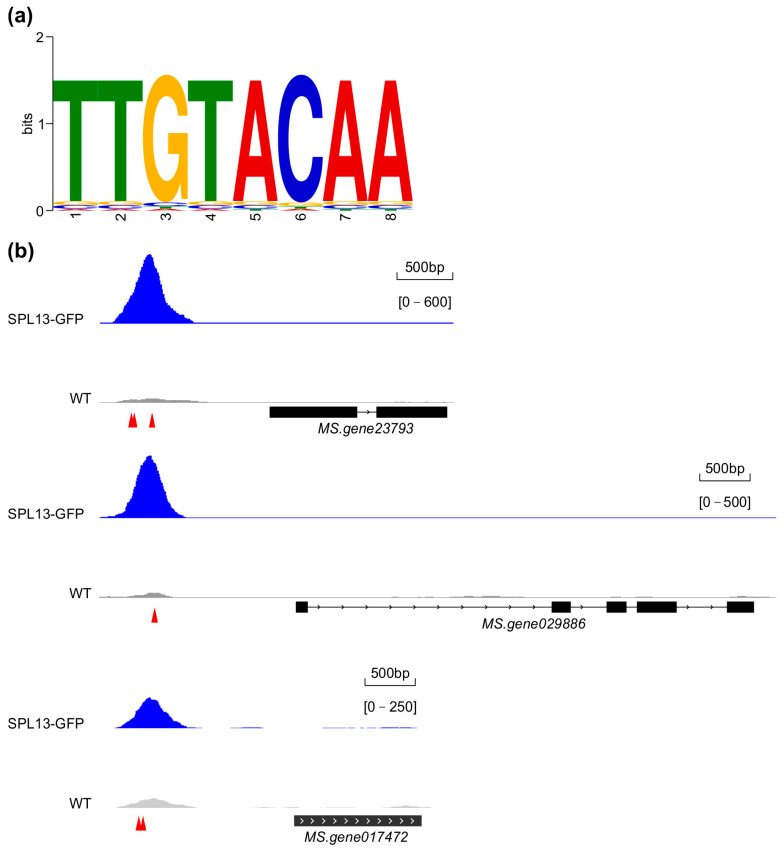
Analysis of SPL13 binding in the genomes of GFP-SPL13 and WT plants. SPL13 binding element (**a**). The occupancy of SPL13 on the prompters of Ms.gene23793, Ms.gene029886, and Ms.gene017472 in SPL13-GFP compared to WT (**b**); the arrows in red indicate the locations of GTAC elements on the promoters of target genes.

To determine the presence of the consensus SPL binding element in the identified regions, a search for the core GTAC element—a critical recognition sequence for SPL13 binding—and a motif enrichment analysis of SPL13 binding sites was performed. The analysis revealed a conserved TTGTACAA element as essential for SPL13 binding (Figure 11a and Table S17 in Supplementary Materials). A comparison of the occupancy of SPL13 on the promoters of three target genes, Ms.gene23793 (cytochrome P450 family), Ms.gene029886 (protein kinase superfamily), and Ms.gene017472 (fasciclin-like arabinogalactan protein), in 35S::*SPL13-GFP* and WT plants (Figure 11b) revealed significantly enhanced SPL13 binding at these promoter regions in 35S::*SPL13-GFP* plants compared to WT (Figure 11b), suggesting a robust regulatory influence of SPL13 on these target genes. This differential occupancy highlights SPL13’s ability to modulate transcription through direct promoter elements, particularly the GTAC one. Together, these findings enhance our understanding of SPL13’s role in transcriptional regulation in alfalfa, demonstrating its precise promoter binding and broader influence on gene expression across the genome.

## 4. Discussion

Aluminum (Al) toxicity in acidic soils severely impairs plant growth, primarily by inhibiting root elongation and lateral root formation, thus limiting nutrient and water uptake [99,100]. Al stress also induces oxidative damage, including the accumulation of reactive oxygen species (ROS) that disrupt cellular functions and membrane integrity [101]. Al toxicity further affects photosynthesis by damaging chloroplasts, reducing pigment levels, and impairing photosystem II (PSII) electron transport [99,100]. MicroRNAs (miRNAs) are key regulators of stress responses [102,103], modulating gene expression post-transcriptionally [104] in plant species such as *Glycine max* [105], *Zea mays* [106,107], and *Arabidopsis thaliana* [108]. miRNA156 (miR156) was the first miRNA to be identified in plants and is known to target SQUAMOSA promoter-binding protein-like (SPL) transcription factors. These SPL transcription factors are integral to various plant developmental processes, including phase transition, floral and architectural development, and fruit formation [109]. The miR156/SPL regulatory module is thus crucial for diverse plant developmental processes, including leaf and fruit development, reproductive transitions, and stress responses [35,110,111,112]. So far, there has been only one report in the literature on the involvement of the miR156/SPL module in the Al response in a plant (barley) [41]. However, the role and mechanism by which miR156/SPL modules regulate Al stress tolerance in alfalfa plants have not been investigated. In this study, we used alfalfa plants with enhanced MsmiR156 expression and plants with reduced or increased expression of SPL13 to investigate the regulatory role of MsmiR156/SPL13 in the Al response in alfalfa. Our findings provide comprehensive insights into the molecular and physiological impacts of MsmiR156 and its target, MsSPL13, on alfalfa’s response to Al stress. The findings elucidate the complex role of miR156 in exacerbating Al toxicity, influencing nutrient uptake, and modulating gene expression, collectively underscoring its importance in the stress physiology of alfalfa.

### 4.1. MsmiR156-OE Exacerbates Al Toxicity in Alfalfa

Our findings demonstrate that *MsmiR156* overexpression significantly worsens Al toxicity in alfalfa roots. Histochemical analyses revealed rapid Al accumulation in *MsmiR156-OE* plants, as indicated by intense hematoxylin staining within 30 min, suggesting heightened sensitivity to Al stress (Figure 1a,d). This sensitivity was further evidenced by increased lipid peroxidation after 4 h and the loss of plasma membrane integrity after 8 h in these transgenic plants compared to WT (Figure 1b,c,e,f). These findings align with previous research in Arabidopsis, where *miR156* overexpression led to higher cadmium (Cd) accumulation in the roots, suggesting a conserved role of miR156 in modulating heavy metal sensitivity across species [113]. Additionally, next-generation sequencing (NGS) studies have identified the Al-induced regulation of miR156 across multiple species. In barley (*Hordeum vulgare*), miR156a and miR156b were significantly upregulated under Al stress [114]. Similarly, in *Medicago truncatula*, miR156g expression increased following Al exposure, further supporting a conserved role of miR156 in Al stress responses [43]. Collectively, these findings suggest that MsmiR156 upregulation amplifies cellular damage under Al stress, potentially by disrupting cellular homeostasis and promoting oxidative stress.

### 4.2. MsmiR156-OE Impairs Nutrient Uptake Under Aluminum Stress

The overexpression of MsmiR156 significantly affected nutrient uptake in alfalfa under Al stress. Our findings revealed a marked decrease in nitrogen, magnesium, and sulfur levels in miR156-OE plants compared to their WT counterparts (Figure 2a–c). This reduction underscores a critical impairment in nutrient absorption, crucial for optimal plant growth and metabolic functions. Notably, while the magnesium levels in MsmiR156-OE plants under Al stress resembled those of WT plants, a significant reduction was evident compared to untreated controls (Figure 2b). This response highlights the complex interplay between miR156 expression and nutrient homeostasis under Al stress, suggesting that miR156 may play a critical role in modulating nutrient absorption pathways in the presence of toxic metal stress [115]. However, it is important to note that our current data demonstrate a correlation between MsmiR156 overexpression and reduced nutrient levels under Al stress, rather than a direct causal relationship. The observed nutrient deficiencies may result from the combined effects of Al toxicity and miR156-mediated alterations in root growth, rather than the direct regulation of nutrient transporters by miR156. These observations are consistent with prior research demonstrating that miR156 regulates various physiological processes, including stress responses. In tobacco, for instance, miR156-OE disrupted mineral nutrient homeostasis, affecting the distribution and levels of several essential minerals, including copper, manganese, zinc, and iron [115]. Similarly, in soybean, miR156 influenced the expression of genes associated with nutrient transport under Al stress, suggesting its broader regulatory role in plant nutrient uptake and homeostasis [42]. Therefore, while our results indicate an association between MsmiR156-OE and impaired nutrient uptake under Al stress, further experiments, such as those examining the direct effects on nutrient transporters, are necessary to determine whether miR156 directly regulates nutrient absorption pathways. The observed nutrient deficiencies in miR156-OE alfalfa plants under Al stress highlight the importance of miR156 in maintaining a nutrient balance and suggest that the targeted manipulation of miR156 expression could potentially alleviate nutrient uptake constraints in crops facing metal toxicity. Future studies investigating the expression of nutrient transporters and associated regulatory pathways in *MsmiR156-OE* plants could provide a deeper understanding of the molecular mechanisms underlying these nutrient deficiencies under Al stress.

### 4.3. MsSPL13 and Root Growth Under Al Stress

Al toxicity in plants is primarily characterized by rapid root growth inhibition [12]. Our study uncovers a novel role for MsSPL13 in regulating root growth under Al stress in alfalfa. Phenotypic analysis of *SPL13-OE* plants showed a significant increase in root length compared to WT plants under Al stress, but silencing *MsSPL13* in *SPL13-RNAi* plants resulted in a substantial root growth reduction (Figure 4b,c), suggesting that *MsSPL13* positively regulates root development in response to Al stress. This effect likely involves the modulation of key stress-responsive pathways responsible for nutrient uptake, root elongation, and cellular protection [12]. These findings indicate that *MsSPL13* plays an essential role in maintaining root growth under Al stress, and its silencing impairs root development, highlighting its critical function in mitigating the negative effects of Al. Furthermore, the contrasting responses between *SPL13-OE* and *SPL13-RNAi* plants provide strong evidence that *MsSPL13* could serve as a key target for genetic manipulation to improve Al tolerance in alfalfa and potentially in other crops exposed to metal stress.

### 4.4. SPL13-Regulated Transcriptomic Changes in Alfalfa Under Al Stress

Our transcriptomic analysis revealed significant gene expression changes in MsSPL13-RNAi alfalfa plants exposed to Al stress, highlighting genotype-specific responses between SPL13-RNAi and WT plants. The principal component analysis (PCA) reinforced this distinction, with the clear clustering of SPL13-RNAi and WT samples, indicating robust differential responses to Al stress (Figure 5a). The genotype-specific clustering in the PCA (18% variance explained) underlines the transcriptional differences between RNAi and WT plants, mirroring the stress response stratification observed in lentil genotypes under Al stress [116]. Notably, the differential expression analysis identified 6149 differentially expressed genes (DEGs), with pronounced transcriptional differences between MsSPL13-RNAi and WT plants under Al stress compared to control conditions (Figure 5b–d). These findings align with previous transcriptomic studies in legumes, including soybean [117] and common bean [118], highlighting the widespread nature of Al-responsive transcriptional changes across plant species. Notably, among the affected genes, key Al detoxification-related genes, namely aluminum-activated malate transporters, were differentially expressed, with one upregulated and another downregulated, while heavy metal-associated domain-containing proteins were downregulated. These findings are in agreement with previous studies, such as in soybean, where Al treatment induced the expression of the AlMT1 malate transporter gene, suggesting a key role for malate exudation in Al detoxification [119]. Similarly, in tomato, the overexpression of Sl-ALMT9 resulted in the differential expression of several malate transporters and related genes, with some members of the *ALMT* and *MATE* families being upregulated and others downregulated, highlighting the complexity of ALMT regulation in response to Al stress [120]. A transcriptome analysis of *Hydrangea macrophylla* identified 11 *ALMT* genes, most of which were upregulated in the stems under Al treatment, further suggesting the involvement of ALMT genes in Al detoxification [121]. Moreover, heavy metal-associated domain-containing proteins, including those in the ATP-binding cassette (ABC) transporter family, are differentially expressed, often being downregulated under metal stress. These proteins play a critical role in metal detoxification and sequestration [122]. These findings support the role of MsSPL13 in regulating Al exclusion mechanisms by influencing the expression of key detoxification genes, highlighting its indirect influence on Al stress tolerance. In contrast, wheat showed a contrasting response, with the upregulation of similar transporters under Al stress [123], emphasizing potential differences in Al detoxification strategies between monocots and dicots. Conversely, genes involved in auxin signaling, such as small auxin-up RNA (SAUR) and auxin-induced proteins, were upregulated in MsSPL13-RNAi plants. This response is consistent with findings in Arabidopsis, where *SAUR55* and *SAUR33* exhibited Al-inducible expression with significant fold changes under stress [91]. Similarly, lentil plants exposed to Al stress showed increased *SAUR23* expression [116], contributing to enhanced root growth plasticity, known as an Al avoidance mechanism [116,124]. The observed increase in auxin-responsive gene expression in RNAi plants suggests a compensatory mechanism that promotes root growth under Al toxicity. Additionally, the downregulation of fasciclin-like arabinogalactan proteins (FLAs)—which are key regulators of cell wall rigidity, altering biochemical properties, cellulose/lignin content, and signaling pathways [125]—suggests that MsSPL13 modulates apoplastic Al exclusion, while reduced cytochrome P450 levels may indicate an altered oxidative stress response—a conserved component of Al tolerance mechanisms across legumes [116,124]. These findings highlight the complex interplay between hormonal signaling, cell wall modification, and detoxification pathways in Al stress responses. Future research should investigate how MsSPL13 interacts with auxin signaling to regulate Al stress tolerance. The functional validation of candidate genes, such as *SAUR* overexpression in root tip mutants, could clarify their role in Al detoxification. Understanding these mechanisms will aid in targeted breeding to develop Al-tolerant alfalfa cultivars.

### 4.5. MsSPL13 Silencing Alters TFs Under Al Stress in Alfalfa

A number of transcription factors (TFs) regulate Al stress responses in plants by activating downstream genes that mediate detoxification and adaptation [96,126,127]. TF families such as WRKY, AP2/ERF, bZIP, NAC, and STOP1 regulate the expression of Al-responsive genes, thereby mediating physiological responses to Al toxicity [96,126,127]. In this study, we found that MsSPL13 played an essential role in regulating these TFs under Al stress. Silencing MsSPL13 led to the downregulation of multiple TF families, including MYB, WRKY, bZIP, C_2_H_2_ zinc finger, NAC, and AP2/ERF (Figure 5e). Notably, MYB transcription factors were significantly repressed in *MsSPL13*-RNAi plants under Al stress. This finding is consistent with observations in *Arabidopsis*, where *MYB103*, an R2R3-type MYB gene, was downregulated in response to Al exposure, leading to altered cell wall properties and increased Al sensitivity due to reduced xyloglucan O-acetylation [128]. In *Arabidopsis*, *MYB103* expression is co-regulated with *TBL27*, a gene involved in xyloglucan O-acetylation, and loss-of-function mutants of *MYB103* exhibited enhanced Al accumulation in the roots and reduced Al tolerance [128]. Similarly, in *Citrus*, species-specific differences in the expression of R2R3-MYB transcription factors under Al stress were evident. For example, the expression of the R2R3-MYB TF and MYB108-like protein was downregulated in *Citrus grandis* roots, while the same TFs were upregulated in *Citrus sinensis* roots [129]. In *Medicago truncatula*, Al-induced microRNAs, such as novel_miR_135 and novel_miR_182, have been shown to suppress MYB target genes at the later stages of Al stress, suggesting a post-transcriptional regulatory mechanism through which microRNAs modulate MYB transcription factor activity under prolonged Al exposure [43]. Conversely, the overexpression of GmMYB183 in Arabidopsis and soybean enhances Al tolerance by enhancing citrate exudation and reducing Al accumulation [130]. MYB TFs regulate Al tolerance through the modulation of the cell wall composition, particularly xyloglucan O-acetylation, and ABA-mediated stress signaling [128]. The consistent repression of MYB TFs in MsSPL13-silenced alfalfa likely compromises cell wall integrity and reduces its detoxification capacity, exacerbating Al toxicity.

In parallel, WRKY TFs were also downregulated in MsSPL13-RNAi plants. This supports prior studies in *M. truncatula*, where miRNA-regulated WRKY genes were repressed under Al stress [43], suggesting a conserved regulatory role in legumes under Al stress. However, in contrast, GmWRKY21 is strongly induced by Al stress in soybean, and its overexpression in Arabidopsis enhances root growth and upregulates key Al tolerance genes, including *ALMT1*, *ALS3*, *MATE*, and *STOP1*, all essential for Al detoxification and transport [95]. This overexpression also led to elevated stress marker expression (KIN1, COR15A/B, RD29A), indicating its key role in managing Al stress responses [95]. Similarly, OsWRKY22 in rice enhances Al tolerance by regulating citrate secretion via OsFRDL4 [131], and AtWRKY47 in Arabidopsis contributes to Al tolerance by modulating cell wall properties through EXTENSIN-LIKE PROTEIN (ELP) and XTH17 [132]. These emphasize the central role of WRKY TFs in Al detoxification, primarily through mechanisms such as organic acid secretion, antioxidant defense, and cell wall remodeling [133,134]. We also observed the downregulation of WRKY53 in MsSPL13-silenced plants, which contrasts previous findings where WRKY53 was upregulated in *Thlaspi caerulescens* under cadmium stress [135]. This suggests that MsSPL13 may modulate miR156-WRKY interactions, as miR156 directly targets SPL13, and its altered expression under Al stress could affect the regulation of downstream *WRKY* genes. The repression of WRKY transcription factors likely contributes to the reduced expression of *ALMT1*, thereby impairing organic acid exudation and compromising root growth under Al toxicity.

We further observed that *MsSPL13* silencing led to the suppression of additional key transcription factor families, including bZIP, C_2_H_2_, NAC, and ERF, each playing a critical role in abiotic stress responses. bZIP proteins are central to ABA signaling and abiotic stress responses [136]. In alfalfa, bZIP transcription factors regulate sucrose signaling and gibberellic acid pathways under low-temperature stress [136]. Their downregulation in MsSPL13-silenced plants likely disrupts ABA-mediated stress adaptation, impairing the activation of antioxidant systems (e.g., glutathione metabolism) and organic acid secretion, key mechanisms for Al detoxification [127,137]. This aligns with findings indicating that bZIPs enhance stress tolerance by modulating ROS homeostasis and ion transport [127,136].

In Arabidopsis, the C_2_H_2_-type zinc-finger transcription factor STOP1 directly regulates the expression of the ALMT1 and MATE genes, which encode malate and citrate transporters, respectively. These transporters play a key role in secreting organic acids that chelate Al^3+^ in the rhizosphere [138,139]. This regulatory mechanism is conserved across plant species, with STOP1/ART1-like proteins controlling Al tolerance genes in both monocots and dicots [139,140]. For instance, studies in Arabidopsis show that STOP1 is essential for Al-induced malate and citrate exudation, and double mutants lacking these transporters exhibited increased Al sensitivity [138,139], suggesting that the downregulation of C_2_H_2_ in *SPL13*-RNAi plants may lead to weakened cell wall integrity, reduced organic acid secretion, and, ultimately, heightened sensitivity to Al stress.

NAC TFs regulate oxidative stress responses and flavonoid biosynthesis, both critical for Al tolerance [136,137,141]. In alfalfa, NAC transcription factors are induced under saline–alkaline stress to enhance ROS scavenging and sucrose metabolism [141]. Their downregulation in MsSPL13-silenced plants likely disrupts redox homeostasis, leading to H_2_O_2_ overaccumulation and impaired flavonoid production, a key mechanism for binding Al^3+^ in root cell walls [142,143]. These results align with transcriptomic studies showing the NAC-mediated activation of phenylpropanoid pathways under acid–Al stress in alfalfa [143].

ERF transcription factors modulate ethylene and auxin signaling under abiotic stress [136,137]. In alfalfa, ERFs are strongly induced during cold stress to regulate glycolysis and citrate cycle pathways [136]. Their suppression in MsSPL13-silenced plants likely disrupts carbon metabolism, reducing energy production (ATP) and organic acid synthesis required for Al chelation [136,142]. Additionally, ERFs interact with MAPK cascades to stabilize ICE1, a regulator of cold-induced CBF genes, suggesting cross-talk between Al and cold stress pathways [136,137]. Together, the downregulation of these transcription factors in MsSPL13-silenced alfalfa highlights MsSPL13’s role as a master regulator of Al-responsive pathways. These findings are consistent with transcriptomic analyses indicating that melatonin-induced Al tolerance in alfalfa relies on similar mechanisms, including cell wall modification and redox homeostasis [142,143]. The silencing of MsSPL13 disrupts a widespread transcriptional network involving bZIP, C_2_H_2_, NAC, and ERF transcription factors, thereby impairing Al exclusion, reactive oxygen species (ROS) scavenging, and metabolic adaptation. This underscores *MsSPL13* as a promising genetic target in improving alfalfa’s resilience in acid soils. We hypothesize that *MsSPL13* acts as a key upstream regulator that brings together hormonal and stress signaling to control transcription factor-driven responses to Al toxicity. Future studies should explore whether *MsSPL13* overexpression enhances Al tolerance by strengthening cell wall integrity, boosting antioxidant defense, and promoting organic acid secretion.

### 4.6. SPL13 Regulates Kinase-Mediated Signaling Under Al Stress

Protein kinases play a crucial role in plant responses to environmental stressors, including Al stress, by activating signal perception, transduction, and downstream defense responses [135,144]. We found that MsSPL13-RNAi alfalfa plants showed the significant downregulation of transcripts encoding protein kinases under Al stress (Figure 7c), suggesting that MsSPL13 contributes to maintaining kinase-mediated signaling during stress. This observation aligns with the broader functional role of SPL genes, which are known to regulate both developmental processes and environmental stress responses in plants [145]. The downregulation of kinase genes in MsSPL13-silenced plants suggests that MsSPL13 may directly or indirectly influence the expression of these signaling components under Al stress conditions. Several protein kinase families are central to Al signaling. For instance, the leucine-rich repeat (LRR) receptor-like kinase aluminum resistance 1 (ALR1) has been identified as a direct Al^3+^ ion sensor in plants [146]. ALR1 initiates a signaling cascade that leads to the activation of NADPH oxidase RbohD, triggering reactive oxygen species (ROS) signaling [146]. This signaling pathway leads to the activation of the STOP1–RAE1 module, enhancing organic acid secretion to detoxify Al in the rhizosphere [146]. Our data suggest that the repression of such kinases in MsSPL13-RNAi plants may limit both intracellular signaling and downstream detoxification mechanisms. Additionally, mitogen-activated protein kinase (MAPK) cascades play crucial roles in regulating plant responses to various environmental stresses, including Al toxicity [144,147]. These MAPK cascades are involved in processes such as signal transduction, the regulation of gene expression, and the activation of stress-responsive proteins [147]. The complex interplay among these kinase-mediated pathways enables plants to perceive Al stress, initiate appropriate cellular responses, and activate adaptive mechanisms to mitigate the harmful effects of Al toxicity [144]. The downregulation of these components in *MsSPL13*-silenced genotypes suggests a reduced ability to trigger rapid and well-organized defense responses to Al stress and increase Al sensitivity.

### 4.7. MsSPL13 Silencing Disrupts a Key Transcriptional Network for Al Detoxification

Al stress activates multiple transcriptional pathways in plants that regulate detoxification, signaling, and root adaptation mechanisms [148]. In this study, the regulatory network model (Figure 9a) showed that silencing *MsSPL13* significantly altered these critical pathways in alfalfa. Notably, *MsSPL13* knockdown led to the downregulation of several key transcription factors, including *AP2/ERF*, *GATA*, *bZIP*, and *STOP1*, all of which are known to regulate abiotic stress signaling and defense gene expression [149,150]. Among these, *STOP1* is a well-established regulator of Al tolerance through its activation of *ALMT1*, which encodes a malate transporter responsible for Al^3+^ chelation and rhizosphere detoxification [149]. The downregulation of both *STOP*1 and *ALMT*1 in MsSPL13-silenced plants suggests a reduced capacity for organic acid-mediated Al exclusion, potentially leading to increased Al sensitivity [149,150]. In addition to transcriptional repression, *MsSPL13* silencing also disrupted ROS signaling, a key early defense pathway that is essential in activating downstream antioxidant and detoxification responses [135,151]. Al stress is known to induce ROS bursts, which activate MAPK cascades and antioxidant enzymes such as SOD and CAT, which together help to limit oxidative damage [152,153]. Impaired ROS signaling in *MsSPL13*-silenced plants likely compromises this protective feedback loop, further weakening the plant’s Al stress response [135,151]. Taken together, these findings indicate that MsSPL13 helps to maintain Al tolerance by supporting the expression of several transcription factors and signaling genes involved in detoxification, including STOP1-mediated malate secretion and ROS scavenging pathways [149,150,151].

### 4.8. SPL13 Directly Binds to Al-Responsive Genes in Alfalfa

Our genome-wide analysis revealed that SPL13 is a key transcriptional regulator in alfalfa, with extensive binding patterns that demonstrate its broad regulatory influence. ChIP-seq analysis revealed *SPL13-GFP* overexpression and identified 8643 binding peaks across 141 genes, a significant increase compared to the 22 peaks associated with 20 genes in WT plants (Figure 10a, Tables S14 and S15). This suggests that elevated SPL13 levels enhance the DNA binding capacity [154,155], potentially by modifying the chromatin structure at target genetic loci, thereby enabling greater SPL13 occupancy. Additionally, the overexpression of SPL13 likely contributes to a broader regulatory impact across various pathways, similar to the effects observed with the Brachypodium bZIP10 (Bradi1g30140) transcription factor [156]. In alfalfa, SPL13 binding sites were predominantly located in promoter regions, accounting for approximately 60% of the peaks, with additional sites found in exons and distal intergenic regions. The proximity of these peaks to transcription start sites (TSSs) emphasizes SPL13’s role in directly regulating gene expression (Figure 10d) [145,157]. The motif enrichment analysis identified the conserved sequence TTGTACAA as essential for SPL13 binding (Figure 11a, Table S17). In 35S::SPL13-GFP plants, enhanced binding was observed at the promoters of key genes, including *cytochrome P450*, *protein kinase*, and *fasciclin-like arabinogalactan* genes, relative to WT plants (Figure 11b). Protein kinases and cytochrome P450 are critical regulators in the Al toxicity response [88,89,90], while FLA18, a fasciclin-like arabinogalactan protein, regulates root growth and cell wall integrity under stress [158]. This suggests a similar role for SPL13 in the Al-induced stress response in alfalfa. These findings are consistent with previous research, where SPL13 was reported to regulate vegetative growth by binding to MYB112 [52] and to promote drought tolerance through direct interaction with *DFR* [157]. In this study, we uncovered a novel mechanism where MsSPL13 directly regulates the expression of genes involved in both growth and stress responses. Specifically, MsSPL13 binds to the promoters of *cytochrome P450*, *protein kinase*, and *fasciclin-like arabinogalactan* genes, downregulating their expression under Al stress (Table S18 in Supplementary Materials). These results suggest that the miR156/SPL13 module plays a critical role in regulating the Al stress response by regulating these genes. Although our ChIP-seq analysis was conducted under control conditions, the RNA-seq data indicated that the expression of these genes is modulated under Al stress, highlighting the dynamic role of SPL13 in stress responses. Future experiments could focus on a time-course ChIP-seq analysis under Al stress to explore the temporal dynamics of SPL13’s binding to its target genes. This would provide valuable insights into how SPL13’s regulatory interactions evolve during stress exposure, enhancing our understanding of its role in the stress response and adaptation in alfalfa.

## 5. Conclusions

MsmiR156 plays a pivotal role in modulating the Al stress response in alfalfa, impacting root integrity, nutrient uptake, and transcriptional regulation. The overexpression of MsmiR156 intensifies Al toxicity by increasing Al accumulation, lipid peroxidation, and membrane damage, leading to severe nutrient deficiencies. At the molecular level, MsmiR156 targets MsSPL13, establishing it as a key post-transcriptional regulator of the Al stress response.

The functional characterization of MsSPL13 revealed its essential role in regulating root growth under Al stress. While MsSPL13-OE plants displayed enhanced root elongation under Al exposure, MsSPL13-RNAi plants showed severe growth inhibition, highlighting SPL13’s importance in Al response mechanisms. Transcriptomic analyses further revealed extensive gene regulatory changes in MsSPL13-RNAi plants, with cytochrome P450s, protein kinases, fasciclin-like arabinogalactan proteins, and aluminum-activated malate transporters significantly downregulated, underscoring the genetic basis of Al susceptibility. ChIP-seq analysis confirmed MsSPL13 directly binds to Al-responsive gene promoters, with strong enrichment at GTAC core motifs, further supporting its role as a key transcriptional regulator. Collectively, these findings establish the MsmiR156–MsSPL13 module as a key regulator of Al response in alfalfa, providing new insights into the genetic networks that govern root adaptation to metal toxicity.

## Data Availability

The datasets generated during and/or analyzed during the current study are available from the corresponding author upon reasonable request.

## Supplementary Materials

The following supporting information can be downloaded at: https://www.mdpi.com/article/10.3390/genes16070751/s1, Figure S1: Differential expression of MsSPL genes in roots of 49-day-old alfalfa plants treated with Al for 0, 8, 24 h; Table S1: Primers used for PCR amplification, genotype analyses, and qRT-PCR; Figure S2: GO terms and functional responses of differentially expressed genes (DEGs) in SPL13-RNAi compared to WT; Table S2: RNA sequencing pipelines and commands; Figure S3: GO term analysis of genotype-specific DEGs in WT and SPL13-RNAi plants under Al stress and control conditions; Table S3: Validation of RNA-seq data using quantitative real-time PCR (qRT-PCR); Table S4: miRNA156d-5p target interactions in *M. sativa*: cleavage sites and alignment; Table S5: Buffers used in ChIP assay and their components; Table S6: ChIP-seq read count and alignment rate; Table S7: ChIP-seq data processing pipelines; Table S8: Summary of RNA-seq read counts and mapping statistics; Table S9: All differentially expressed genes (DEGs) in SPL13-RNAi and WT plants under both aluminum stress and control conditions; Table S10: Differentially expressed transcription factors (DETFs) in SPL13-RNAi and WT plants under both aluminum stress and control conditions; Table S11: GO enrichment and functional categorization of differentially expressed genes (DEGs) in SPL13-RNAi compared to WT under aluminum stress and control conditions; Table S12: Genotype-specific comparison of differentially expressed genes (DEGs) between SPL13-RNAi and WT plants under aluminum stress and control conditions; Table S13: Analysis of the SPL13 gene regulatory network under Al stress; Table S14: Number of genes in SPL13-GFP and WT plants; Table S15: Peak number in SPL13-GFP and WT plants; Table S16: Pie chart of the distribution of SPL13 peaks in the downstream region, distal intergenic region, exons, introns, and promoters in the genome; Table S17: Peak position with GTAC in SPL13-GFP and WT plants; Table S18: Genes directly regulated by SPL13 under Al stress in SPL13-RNAi and WT plants.

## Abbreviations

The following abbreviations are used in this manuscript:

Al	Aluminum
miRNA156 (miR156)	MicroRNA156
SPL13	SQUAMOSA promoter-binding protein-like 13
ChIP-seq	Chromatin immunoprecipitation sequencing
WT	Wild type
GRAS	Gibberellic acid insensitive, repressor of GAI, and scarecrow
Myb-related proteins	Myb transcription factors
bHLH041	Basic helix–loop–helix 041
NAC	NAC domain-containing transcription factors
WRKY53	WRKY transcription factor 53
bZIP	Basic leucine zipper
AGAMOUS-like MADS-box	AGAMOUS-like MADS-box transcription factors
RT-qPCR	Reverse transcription quantitative PCR
FASTQ	Sequence file format for high-throughput sequencing data
PCA	Principal component analysis
DEGs	Differentially expressed genes
GO	Gene Ontology
FIMO	Find individual motif occurrences
SEA	Single element analysis
MEME	Multiple EM for Motif Elicitation
RISC	RNA-induced silencing complex
HPC	High-performance computing
RNA-seq	RNA sequencing
DE	Differentially expressed
MADS-box	MADS-domain transcription factors
LRR	Leucine-rich repeat
ABC	ATP-binding cassette
SAUR	Small auxin-up RNA
TCA	Tricarboxylic acid cycle
PSII	Photosystem II
ROS	Reactive oxygen species
SPL	SQUAMOSA promoter-binding protein-like
STOP1	Stress-responsive element binding protein 1
GTAC	Core motif in SPL binding
ACC1	Acetyl-CoA carboxylase 1
ACC2	Acetyl-CoA carboxylase 2
Bp	Base pair
cDNA	Complementary DNA
DETF(s)	Differentially expressed transcription factor(s)
FLA	Fasciclin-like arabinogalactan protein
h	Hour(s)
HPC	High-performance computing
ICP-OES	Inductively coupled plasma optical emission spectroscopy
PCR	Polymerase chain reaction
psRNATarget	Plant small RNA target analysis
SEM	Standard error of the mean
TCA	Tricarboxylic acid cycle
TSS	Transcription start site
TF(s)	Transcription factor(s)
Tris-HCl	Tris(hydroxymethyl)aminomethane hydrochloride
UTR	Untranslated region
