# Supplementary material for: Aluminum Stress Response Is Regulated Through a miR156/SPL13 Module in Medicago sativa
Source: Genes (Basel). 2025 Jun 27;16(7):751. doi: 10.3390/genes16070751 (PMC12295421; doi:10.3390/genes16070751)
Supplement: Supplementary file 1 [file genes-16-00751-s001.zip › June 25_25_Supplemental Figures and Tables.docx]

**Aluminum Stress Response Is Regulated Through a miR156/SPL13 Module in *Medicago sativa***

**Gamalat Allam¹˒², Solihu K. Sakariyahu****¹˒², Binghui Shan¹˒², Banyar Aung², Tim McDowell¹, Yousef Papadopoulos³, Mark A. Bernards², Abdelali Hannoufa¹˒²^,^***

Supplemental Data

**Table S1**. Primers used for PCR amplification, genotype analyses and qRT-PCR**.**  Asterisks (*) indicate the references genes used for data normalization based on (Alexander et al. 2007).

| **Name** | **Sequence (5'-3')** | **Amplicon size (bp** | **Target gene** |
| --- | --- | --- | --- |
| OEMsSPL13-F | CACCATGGAGTGGAATTTGAAAGC | 1135 | *SPL*13 |
| OEMsSPL13-R | CTATTCCCATTGATAGGGAAATAGT | 1135 | *SPL*13 |
| 35S-F3 | CAATCCCACTATCCTTCGCAAGACCC | 1300 | 35S Promoter |
| pMDC32 control | TCATCAGGCGGGCAAGAATGTGAATA | 1300 | pMDC32 vector |
| Ms-SPL13Fq1 | ACTGCAACCACCTACGAAGA | 93 | *SPL13* |
| Ms-SPL13Rq1 | AAGAAGAAGTGTGGTTTGGCT | 93 | *SPL13* |
| Ms-SPL13a-Fq1 | CTTGGGTTGGAGGAGATGTT | 100 | *SPL13a* |
| Ms-SPL13a-Rq1 | GCTCTGGTTGAAGATGTTG | 100 | *SPL13a* |
| Ms-SPL12Fq1 | CCTCAGCCTGAAGCAGTGAA | 174 | *SPL12* |
| Ms-SPL12Rq1 | CTTGCTGTTGGGCATGTCTG | 174 | *SPL12* |
| Ms_156Fq3 | TGTTCCCATTCATCACCTCCAA | 80 | *MsmiR156* |
| Ms_156Rq3 | AGGGAGTAGCGGTGATCTTG | 80 | *MsmiR156* |
| Acc1-F* | GATCAGTGAACTTCGCAAAGTAC | 91 | *Acetyl CoA* |
| Acc1-R* | CAACGACGTGAACACTACAAC | 91 | *Acetyl CoA* |
| Acc2-F* | GATCAGTGAACTTCGCAAAGTAC | 154 | *Acetyl CoA* |
| Acc2-R* | GAGGGATGCTGCTACTTTGATG | 154 | *Acetyl CoA* |
| LA-MsSPL9-Fq1 | AGATACAGCTCTTGCTACTG | 112 | *SPL9* |
| LA-MsSPL9-Rq1 | GTTGAGAATGTTGAACTGAC | 112 | *SPL9* |
| Ms-SPL6Fq1 | CTCGGCCGATACATCAAACTG | 191 | *SPL6* |
| Ms-SPL6Rq1 | CCTCTGTTCAACACCATTGACG | 191 | *SPL6* |
| MsSPL7a-Fq1 | CAGGGCACAATGAGAGAAGA | 130 | *SPL7a* |
| MsSPL7a-Rq1 | GCCATGAATCAGTCCGAGAT | 130 | *SPL7a* |
| MsSPL8-Fq1 | TGGCCGCACTTACTTCTCTT | 100 | *SPL8* |
| MsSPL8-Rq1 | AACCTTCGGCTTGACACCTA | 100 | *SPL8* |
| LA-MsSPL4-Fq1 | GATGACTCAAAAAGAAGTTG | 97 | *SPL4* |
| LA-MsSPL4-Rq1 | ATGTCTGAAATCATTCTCCT | 97 | *SPL4* |
| MsSPL3-Fq1 | GCTTGCAGAGTTTGATGAATCG | 112 | *SPL3* |
| MsSPL3-Rq1 | GCCAGTACCGCTTCCTTCAG | 112 | *SPL3* |
| MsSPL2a-Fq1 | GCTGCAGACGACAACTTTCA | 100 | *SPL2* |
| MsSPL2a-Rq1 | CATCTGTTGCTTCCCATCAC | 100 | *SPL2* |
| MsSPL11-Fq1 | CTTGTCTTGTGGATGGGTGC | 147 | *SPL11* |
| MsSPL11-Rq1 | GAAACCTGCTACACTGTTGGC | 147 | *SPL11* |

**Table S2. RNA Sequencing Pipelines and Commands**

| Step | Description and Commands |
| --- | --- |
| Sequencing | Paired-end sequencing of raw reads (fastq files) performed using |
| Method | Illumina sequencing platforms. |
| Computational Resource | Analyses were performed using computational resources provided by the Digital Research Alliance of Canada HPC cluster |
| Quality Control (FastQC) | module load fastqc  for file in *.fq.gz  do fastqc $file  done |
| Adaptor and Quality Trimming (Trimmomatic) | Parameters and commands used for adaptor removal and quality trimming  module load java  for file in *_1.fq.gz  do base=$(basename ${file} _1.fq.gz)  module load trimmomatic  java -jar $EBROOTTRIMMOMATIC/trimmomatic-0.39.jar PE -threads 20 -phred33 ${file} ${base}_2.fq.gz ${base}_R1_paired.fastq ${base}_R1_unpaired.fastq ${base}_R2_paired.fastq ${base}_R2_unpaired.fastq ILLUMINACLIP:adapters.fa:2:30:10 HEADCROP:4 LEADING:20 TRAILING:20 SLIDINGWINDOW:4:15 MINLEN:40 |
| Alignment and mapping of the reads with the reference genome (Hisat2) | Parameters –  module load hisat2  hisat2-build -p 8 -f final_genome.fasta referenceGenome_Ms  module load hisat2  for file in *_R1_paired.fastq  do base=$(basename ${file} _R1_paired.fastq)  hisat2 -p 20 --dta --phred33 -S $file.sam -x referenceGenome_Ms -1 $file -2 ${base}_R2_paired.fastq -U ${base}_R1_unpaired.fastq,${base}_R2_unpaired.fastq  Reference genome of Medicago sativa from Figshare (<https://figshare.com/projects/whole_genome_sequencing_and_assembly_of_Medicago_sativa/66380>) |
| Quantifying the gene counts (featureCounts) | Parameters –  module load StdEnv/2020  module load gcc/9.3.0  module load subread  module load gffread  gffread -E -T Mtruncatula_285_Mt4.gff3 -o Mtruncatula_285_Mt4.gtf  featureCounts -p -T 16 -s 2 -a Mtruncatula_285_Mt4.gft -o GeneCounts.txt *.sam |

**
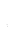

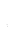

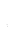
Table S3.** Validation of RNA-seq data using quantitative real-time PCR (qRT-PCR)



| **Gene name** | **SPL13-RNAi-05/WT** | | **SPL13-RNAi-06/WT** | |
| --- | --- | --- | --- | --- |
|  | **RNA-seq** | **qRT-PCR** | **RNA-seq** | **qRT-PCR** |
| Protein kinase | - 2.28 | -2.10 | -1.98 | -1.88 |
| Fasciclin-like arabinogalactan protein | - 0.90 | - 0.88 | - 0.75 | - 0.68 |
| Cytochrome P450 | - 4.0 | -3.8 | - 3.02 | - 2.10 |
| Aluminum-activated malate transporter | -1.20 | - 1.07 | -1.10 | - 0.98 |
| Myb-related protein Myb4-like | - 4.08 | - 3.5 | -3.2 | - 2.10 |
| WRKY transcription factor 53 | - 2.85 | - 1.90 | - 1.88 | - 1.50 |
| GRAS family transcription factor | 2.63 | 2.10 | 2.40 | 1.98 |
| Transcription factor bHLH041 | 5.76 | 4.60 | 3.89 | 2.47 |
| NAC domain-containing protein | 3.21 | 3.20 | 2.86 | 2.16 |
| Transcription factor bZIP-1 | 4.39 | 4.20 | 3.17 | 2.90 |
| Agamous-like MADS-box protein AGL9 homolog | 21.39 | 18.24 | 16.20 | 14.70 |

(-) Negative values indicate downregulation, whereas positive values indicate upregulation relative to WT. RNA-seq and qRT-PCR results showed consistent expression patterns, confirming the reliability of the RNA-seq data.

**Table S4:** see Supplementary Sequencing Data

**
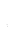

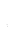

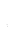
****Table S5.** Buffers Used in ChIP Assay and Their Components



| \| **Buffers** \|  \|  \| \| --- \| --- \| --- \| | **Chemicals** | **Concentration** |
| --- | --- | --- | --- | --- | --- |
| **Extraction buffer S** | HEPES-KOH pH 7.5 | 50 mM |
|  | NaCl | 150 mM |
|  | EDTA | 1 mM |
|  | Triton X-100 | 1% |
|  | sodium deoxycholate | 0.1% |
|  | SDS | 1% |
| **Extraction buffer F** | HEPES-KOH pH 7.5 | 50 mM |
|  | NaCl | 150 mM |
|  | EDTA | 1 mM |
|  | Triton X-100  Sodium deoxycholate | 1%  0.1% |

| **High salt wash buffer** | SDS | 0.1% |
| --- | --- | --- |
|  | Triton X-100 | 1% |
|  | EDTA | 2 mM |
|  | Tris-HCl pH=8) | 20 mM |
|  | NaCl | 500 mM |
| **Low salt wash buffer** | SDS | 0.1% |
|  | Triton X-100 | 1% |
|  | EDTA | 2 mM |
|  | Tris-HCl (pH=8) | 20 mM |
|  | NaCl | 150 mM |
| **LiCl wash buffer** | LiCl | 0.25 M |
|  | NP-40 | 1% |
|  | Sodium deoxycholate | 1% |
|  | EDTA | 1 mM |
|  | Tris-HCl (pH=8) | 10 mM |
| **Elution buffer** | SDS | 10 % |
|  | NaHCO3 | 1M |

| TE buffer | EDTA | 1 mM |
| --- | --- | --- |
|  | Tris-HCl (pH=8) | 10 mM |

**Table S6:** ChIP-seq Read Count and Alignment Rate

| **Sample name** | **Total read count** | **Alignment rate (%)** |
| --- | --- | --- |
| WT1 | 68621586 | 66.49% |
| WT2 | 84036114 | 46.16% |
| WT-Input | 110281789 | 88.39% |
| SPL13-1 | 51571173 | 77.96% |
| SPL13-2 | 75606871 | 59.42% |
| SPL13-Input | 69269767 | 93.30% |

**Table S7.** ChIP-seq Data Processing Pipelines

| Step | Command |
| --- | --- |
| Sequencing | Sequenced for paired-end raw reads (fastq files) |
| Method | Illumina sequencing platforms |
| Use Bowtie2 to map raw reads to the Medicago sativa genome | bowtie2 –p 4 -x index folder -1 *_1.fastq -2 *_2.fastq -S *.sam 2> mapping_stats.txt |
| Use Picard to remove duplicated reads | java -jar -Xmx15g -XX:ParallelGCThreads=6 picard.jar MarkDuplicates \  INPUT=input.bam \  OUTPUT=output.bam \  METRICS_FILE=metrics.metric \  REMOVE_DUPLICATES=true |
| Use MACS2 to identigy enriched region (peaks) | macs2 callpeak -t sample.bam -c input.bam -f BAMPE -g 1864511547 --nomodel --extsize 200 --shift 0 -B --qvalue 0.05 --bw 300 -n folder |
| Use bamCoverage in deepTools to converted the peaks to bigwig files | bamCoverage --bam *.bam \  -o *.bam.bw \  --binSize 10 --normalizeUsing RPKM \  --effectiveGenomeSize 1864511547 |
| Use Integrative Genomics Viewer (IGV) to visualize the peak files | |





**Figure S1**. Differential expression *MsSPL* genes in roots of 49-day-old alfalfa plants treated with Al for 0, 8, 24 hrs. Each bar plot represents the mean. Error bars represent (SEM). A one-way ANOVA was conducted with n = 4 individual plants. Significant differences detected from the one-way ANOVA in R (version R-4.2.3) were followed by a post hoc Tukey multiple comparison test. Means with the same letters are not significantly different at *p* ≤ (value).

**Table S8**. Summary of RNA-seq Read Counts and Mapping Statistics

| **Sample** | **Raw reads** | **Clean reads** | **% Mapped reads** | **Gene assignment** |
| --- | --- | --- | --- | --- |
| **Al1WT1** | **69516131** | **68276177** | **91.39** | **44118544 (15.2%)** |
| **AL2WT4** | **63206914** | **62069700** | **93.2** | **40148923 (15.1%)** |
| **AL3WT5** | **72760424** | **72405401** | **92.94** | **49068448 (16.3%)** |
| **AL413051** | **62747605** | **61711299** | **93.01** | **40902612 (15.6%)** |
| **AL513054** | **68842207** | **67739685** | **93.05** | **45623052 (16.0%)** |
| **AL613053** | **62963220** | **62017332** | **92.88** | **41441515 (15.8%)** |
| **AL713061** | **69833888** | **68683174** | **93.3** | **46430492 (15.9%)** |
| **AL813063** | **59658779** | **58621617** | **93.05** | **38001481 (15.1%)** |
| **AL913064** | **62851021** | **61682068** | **92.78** | **39745632 (15.0%)** |
| **C1WT4** | **65327188** | **64255127** | **93.28** | **41526145 (15.1%)** |
| **C2WT5** | **61780217** | **60325668** | **93.24** | **37589817 (14.1%)** |
| **C3WT6** | **61684264** | **60612479** | **92.78** | **40687559 (15.8%)** |
| C413053 | **61302682** | **60323684** | **92.71** | **39399745 (15.5%)** |
| **C513051** | **64617994** | **63424721** | **92.14** | **40448134 (14.9%)** |
| **C613054** | **72796087** | **71587592** | **92.92** | **47081689 (15.6%)** |
| **C713061** | **71512642** | **70383923** | **93.4** | **47194857 (15.8%)** |
| **C813062** | **64492954** | **63224904** | **92.94** | **40378851 (14.8%)** |
| **C913063** | **66517413** | **65310389** | **92.26** | **41731786 (15.1%)** |
| **Total** | **1,182,411,630** | **1,162,654,940**  **( 98.33%)** |  | **761519282** |
| **Average** | **65,689,535** | **64,591,941.11** | **92.85** | **42,306,626.78** |
| **Average WTC** |  | **61,731,091.33** |  | **39,934,507** |
| **Average SPL13RNAiC** |  | **65,709,202.17** |  | **42,705,843.67** |
| **Average WT Al** |  | **67,583,759.33** |  | 44,445,305 |
| **Average SPL13RNAi Al** |  | **63,409,195.83** |  | 42,024,130.67 |

**
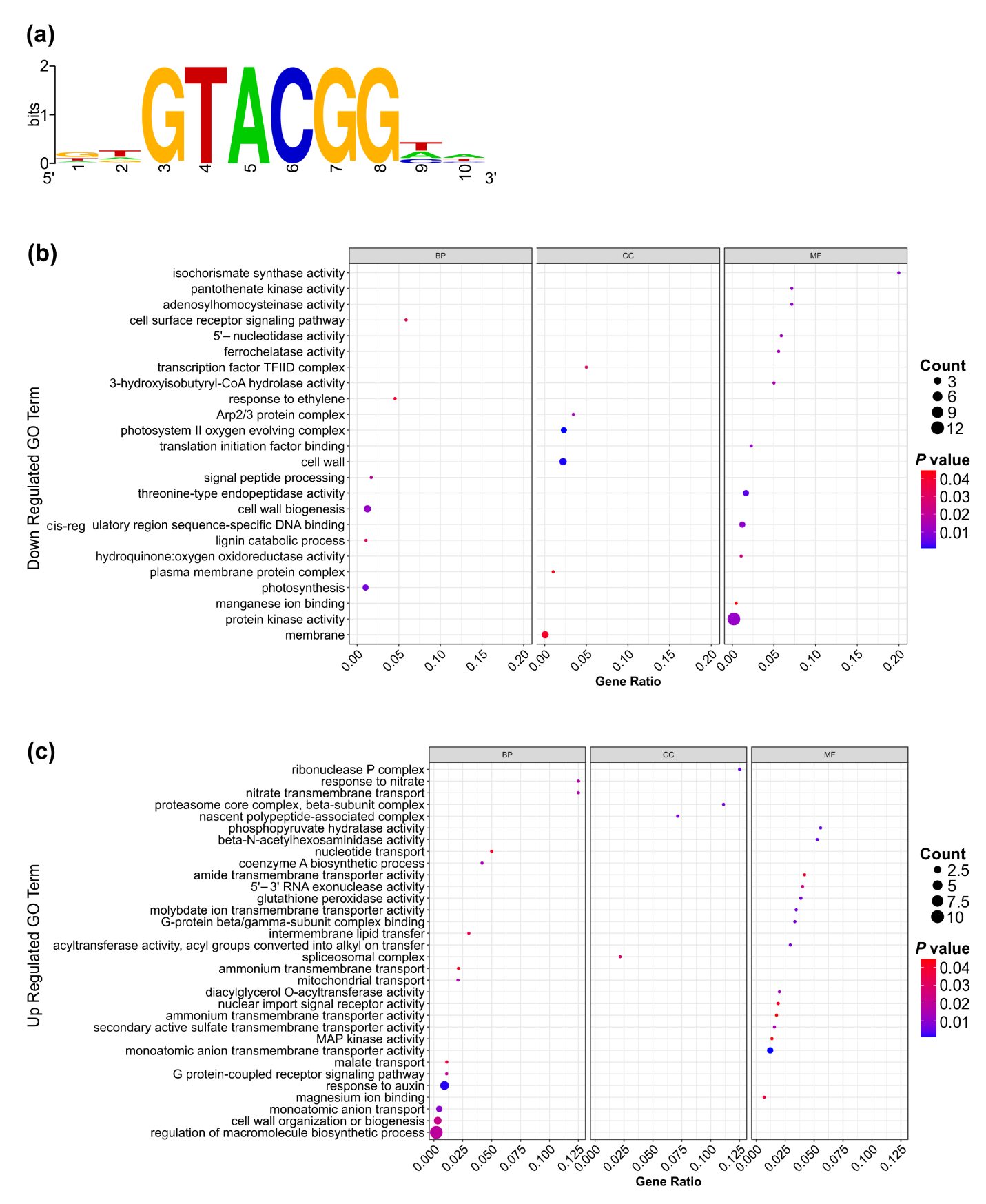
**

**Figure S2**. GO term and functional response of differentially expressed genes (DEGs) in SPL13-RNAi compared to WT. Enrichment of the core GTAC motif in the promoter of all DEGs (**a**), Dot plot representing the enriched GO terms for CC,BP, and MF among the downregulated DEGs (**b**). Dot plot showing the enriched GO terms for CC,BP, and MF among the upregulated DEGs (**c**)


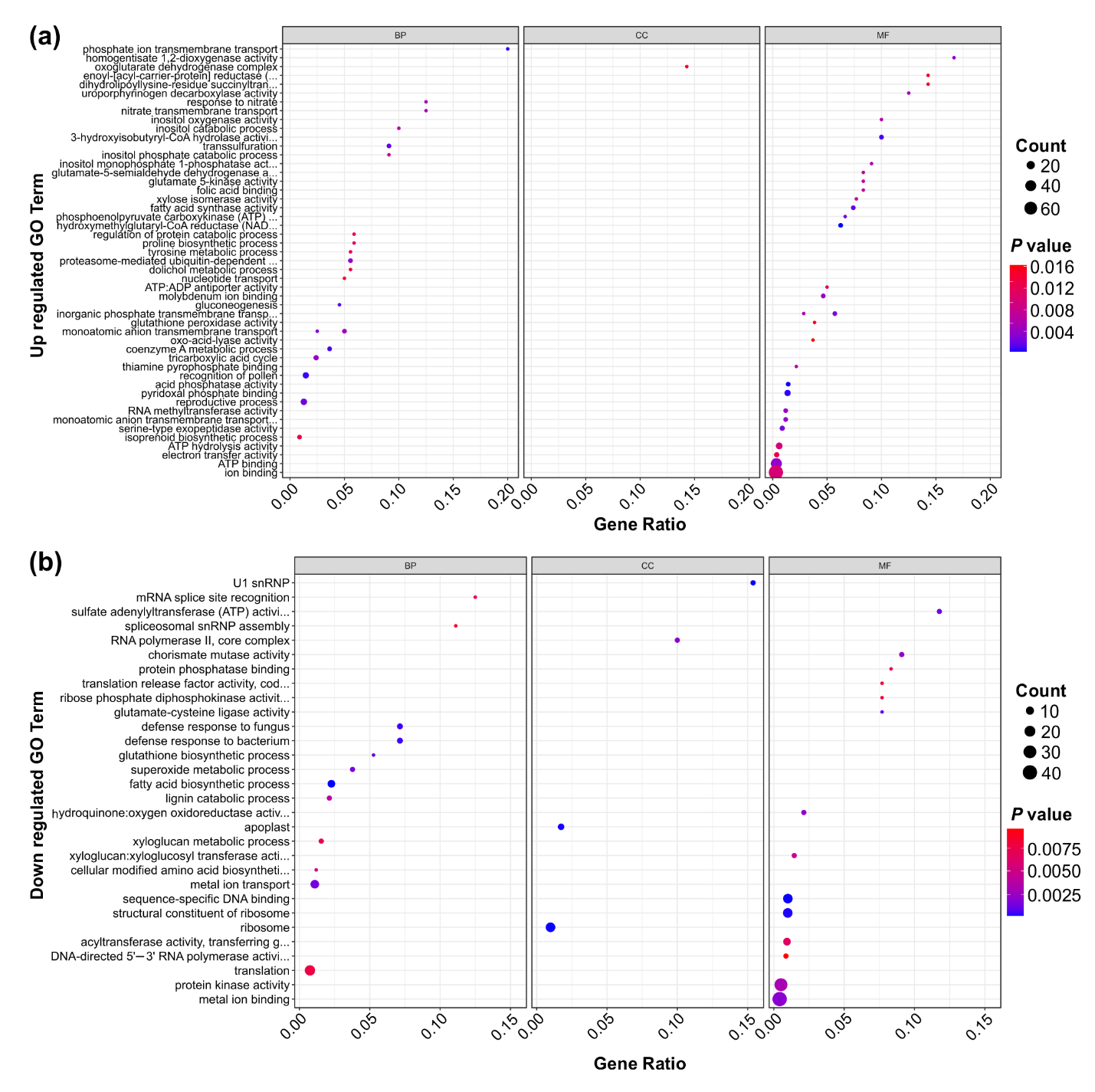


**Figure S3**. GO-term analysis of genotype-specific comparison of DEGs in WT and SPL13RNAi plants under Al stress and control conditions. Dot plot depicting the enriched GO terms for upregulated DEGs (a). Dot plot depicting the enriched GO term for downregulated DEGs (b).

**Tables S9-S18**: see Supplementary Sequencing Data
