# Supplementary material for: Aluminum Stress Response Is Regulated Through a miR156/SPL13 Module in Medicago sativa
Source: Genes (Basel). 2025 Jun 27;16(7):751. doi: 10.3390/genes16070751 (PMC12295421; doi:10.3390/genes16070751)
Supplement: Supplementary file 1 [file genes-16-00751-s001.zip › June 25_25_Supplemental Figures and Tables.pdf]

Aluminum Stress Response Is Regulated Through a miR156/SPL13 Module in *Medicago sativa*

Gamalat Allam[1,2], Solihu K. Sakariyahu[1,2], Binghui Shan[1,2], Banyar Aung[2], Tim McDowell[1], Yousef Papadopoulos[3], Mark A. Bernards[2], Abdelali Hannoufa[1,2,*]

Supplemental Data

Table S1. Primers used for PCR amplification, genotype analyses and qRT-PCR. Asterisks (*) indicate the references genes used for data normalization based on (Alexander et al. 2007).

Name	Sequence (5'-3')	Amplicon size (bp	Target gene
OEMsSPL13-F	CACCATGGAGTGGAATTTGAAAGC	1135	*SPL*13
OEMsSPL13-R	CTATTCCCATTGATAGGGAAATAGT	1135	*SPL*13
35S-F3	CAATCCCACTATCCTTCGCAAGACCC	1300	35S Promoter
pMDC32 control	TCATCAGGCGGGCAAGAATGTGAATA	1300	pMDC32 vector
Ms-SPL13Fq1	ACTGCAACCACCTACGAAGA	93	*SPL13*
Ms-SPL13Rq1	AAGAAGAAGTGTGGTTTGGCT	93	*SPL13*
Ms-SPL13a-Fq1	CTTGGGTTGGAGGAGATGTT	100	*SPL13a*
Ms-SPL13a-Rq1	GCTCTGGTTGAAGATGTTG	100	*SPL13a*
Ms-SPL12Fq1	CCTCAGCCTGAAGCAGTGAA	174	*SPL12*
Ms-SPL12Rq1	CTTGCTGTTGGGCATGTCTG	174	*SPL12*
Ms_156Fq3	TGTTCCCATTCATCACCTCCAA	80	*MsmiR156*
Ms_156Rq3	AGGGAGTAGCGGTGATCTTG	80	*MsmiR156*
Acc1-F*	GATCAGTGAACTTCGCAAAGTAC	91	*Acetyl CoA*
Acc1-R*	CAACGACGTGAACACTACAAC	91	*Acetyl CoA*
Acc2-F*	GATCAGTGAACTTCGCAAAGTAC	154	*Acetyl CoA*
Acc2-R*	GAGGGATGCTGCTACTTTGATG	154	*Acetyl CoA*
LA-MsSPL9-Fq1	AGATACAGCTCTTGCTACTG	112	*SPL9*
LA-MsSPL9-Rq1	GTTGAGAATGTTGAACTGAC	112	*SPL9*
Ms-SPL6Fq1	CTCGGCCGATACATCAAACTG	191	*SPL6*
Ms-SPL6Rq1	CCTCTGTTCAACACCATTGACG	191	*SPL6*

MsSPL7a-Fq1	CAGGGCACAATGAGAGAAGA	130	*SPL7a*
MsSPL7a-Rq1	GCCATGAATCAGTCCGAGAT	130	*SPL7a*
MsSPL8-Fq1	TGGCCGCACTTACTTCTCTT	100	*SPL8*
MsSPL8-Rq1	AACCTTCGGCTTGACACCTA	100	*SPL8*
LA-MsSPL4-Fq1	GATGACTCAAAAAGAAGTTG	97	*SPL4*
LA-MsSPL4-Rq1	ATGTCTGAAATCATTCTCCT	97	*SPL4*
MsSPL3-Fq1	GCTTGCAGAGTTTGATGAATCG	112	*SPL3*
MsSPL3-Rq1	GCCAGTACCGCTTCCTTCAG	112	*SPL3*
MsSPL2a-Fq1	GCTGCAGACGACAACTTTCA	100	*SPL2*
MsSPL2a-Rq1	CATCTGTTGCTTCCCATCAC	100	*SPL2*
MsSPL11-Fq1	CTTGTCTTGTGGATGGGTGC	147	*SPL11*
MsSPL11-Rq1	GAAACCTGCTACACTGTTGGC	147	*SPL11*

Table S2. RNA Sequencing Pipelines and Commands

Step	Description and Commands
Sequencing	Paired-end sequencing of raw reads (fastq files) performed using
Method	Illumina sequencing platforms.
Computational Resource	Analyses were performed using computational resources provided by the Digital Research Alliance of Canada HPC cluster
Quality Control (FastQC)	module load fastqc<br>for file in *.fq.gz<br>do fastqc $file<br>done
Adaptor and Quality Trimming (Trimmomatic)	Parameters and commands used for adaptor removal and quality trimming<br>module load java<br>for file in *_1.fq.gz<br>do base=$(basename ${file} _1.fq.gz)<br>module load trimmomatic

	java -jar $EBROOTTRIMMOMATIC/trimmomatic-0.39.jar PE -threads 20 -phred33 ${file} ${base}_2.fq.gz ${base}_R1_paired.fastq ${base}_R1_unpaired.fastq ${base}_R2_paired.fastq ${base}_R2_unpaired.fastq ILLUMINACLIP:adapters.fa:2:30:10 HEADCROP:4 LEADING:20 TRAILING:20 SLIDINGWINDOW:4:15 MINLEN:40
Alignment and mapping of the reads with the reference genome (Hisat2)	Parameters – <br>module load hisat2 <br>hisat2-build -p 8 -f final_genome.fasta referenceGenome_Ms <br>module load hisat2 <br>for file in *_R1_paired.fastq <br>do base=$(basename ${file} _R1_paired.fastq) <br>hisat2 -p 20 --dta --phred33 -S $file.sam -x referenceGenome_Ms -1 $file -2 ${base}_R2_paired.fastq -U ${base}_R1_unpaired.fastq,${base}_R2_unpaired.fastq <br>Reference genome of Medicago sativa from Figshare (https://figshare.com/projects/whole_genome_sequencing_and_assembly_of_Medicago_sativa/66380)
Quantifying the gene counts (featureCounts)	Parameters – <br>module load StdEnv/2020 <br>module load gcc/9.3.0 <br>module load subread <br>module load gffread <br>gffread -E -T Mtruncatula_285_Mt4.gff3 -o Mtruncatula_285_Mt4.gtf <br>featureCounts -p -T 16 -s 2 -a Mtruncatula_285_Mt4.gft -o GeneCounts.txt *.sam

Table S3. Validation of RNA-seq data using quantitative real-time PCR (qRT-PCR)

**Gene name**	**SPL13-RNAi-05/WT**		**SPL13-RNAi-06/WT**	
	**RNA-seq**	**qRT-PCR**	**RNA-seq**	**qRT-PCR**
Protein kinase	- 2.28	-2.10	-1.98	-1.88

.

Fasciclin-like arabinogalactan protein	- 0.90	- 0.88	- 0.75	- 0.68
Cytochrome P450	- 4.0	-3.8	- 3.02	- 2.10
Aluminum-activated malate transporter	-1.20	- 1.07	-1.10	- 0.98
Myb-related protein Myb4-like	- 4.08	- 3.5	-3.2	- 2.10
WRKY transcription factor 53	- 2.85	- 1.90	- 1.88	- 1.50
GRAS family transcription factor	2.63	2.10	2.40	1.98
Transcription factor bHLH041	5.76	4.60	3.89	2.47
NAC domain-containing protein	3.21	3.20	2.86	2.16
Transcription factor bZIP-1	4.39	4.20	3.17	2.90
Agamous-like MADS-box protein AGL9 homolog	21.39	18.24	16.20	14.70

(-) Negative values indicate downregulation, whereas positive values indicate upregulation relative to WT. RNA-seq and qRT-PCR results showed consistent expression patterns, confirming the reliability of the RNA-seq data.

Table S4: see Supplementary Sequencing Data

Table S5. Buffers Used in ChIP Assay and Their Components

**Buffers**	**Chemicals**	**Concentration**
**Extraction buffer S**	HEPES-KOH pH 7.5	50 mM
	NaCl	150 mM
	EDTA	1 mM
	Triton X-100	1%
	sodium deoxycholate	0.1%
	SDS	1%
**Extraction buffer F**	HEPES-KOH pH 7.5	50 mM
	NaCl	150 mM
	EDTA	1 mM
	Triton X-100	1%

	Sodium deoxycholate	0.1%
**High salt wash buffer**	SDS	0.1%
	Triton X-100	1%
	EDTA	2 mM
	Tris-HCl pH=8)	20 mM
	NaCl	500 mM
**Low salt wash buffer**	SDS	0.1%
	Triton X-100	1%
	EDTA	2 mM
	Tris-HCl (pH=8)	20 mM
	NaCl	150 mM
**LiCl wash buffer**	LiCl	0.25 M
	NP-40	1%
	Sodium deoxycholate	1%
	EDTA	1 mM
	Tris-HCl (pH=8)	10 mM
**Elution buffer**	SDS	10 %
	NaHCO3	1M
TE buffer	EDTA	1 mM
	Tris-HCl (pH=8)	10 mM

Table S6: ChIP-seq Read Count and Alignment Rate

Sample name	Total read count	Alignment rate (%)
WT1	68621586	66.49%
WT2	84036114	46.16%
WT-Input	110281789	88.39%
SPL13-1	51571173	77.96%
SPL13-2	75606871	59.42%
SPL13-Input	69269767	93.30%

Table S7. ChIP-seq Data Processing Pipelines

Step	Command
Sequencing	Sequenced for paired-end raw reads (fastq files)
Method	Illumina sequencing platforms
Use Bowtie2 to map raw reads to the Medicago sativa genome	bowtie2 –p 4 -x index folder -1 *_1.fastq -2 *_2.fastq -S *.sam 2> mapping_stats.txt
Use Picard to remove duplicated reads	java -jar -Xmx15g -XX:ParallelGCThreads=6 picard.jar MarkDuplicates \ INPUT=input.bam \ OUTPUT=output.bam \ METRICS_FILE=metrics.metric \ REMOVE_DUPLICATES=true
Use MACS2 to identigy enriched region (peaks)	macs2 callpeak -t sample.bam -c input.bam -f BAMPE -g 1864511547 --nomodel --extsize 200 --shift 0 -B --qvalue 0.05 --bw 300 -n folder
Use bamCoverage in deepTools to converted the peaks to bigwig files	bamCoverage --bam *.bam \ -o *.bam.bw \ --binSize 10 --normalizeUsing RPKM \ --effectiveGenomeSize 1864511547
Use Integrative Genomics Viewer (IGV) to visualize the peak files	

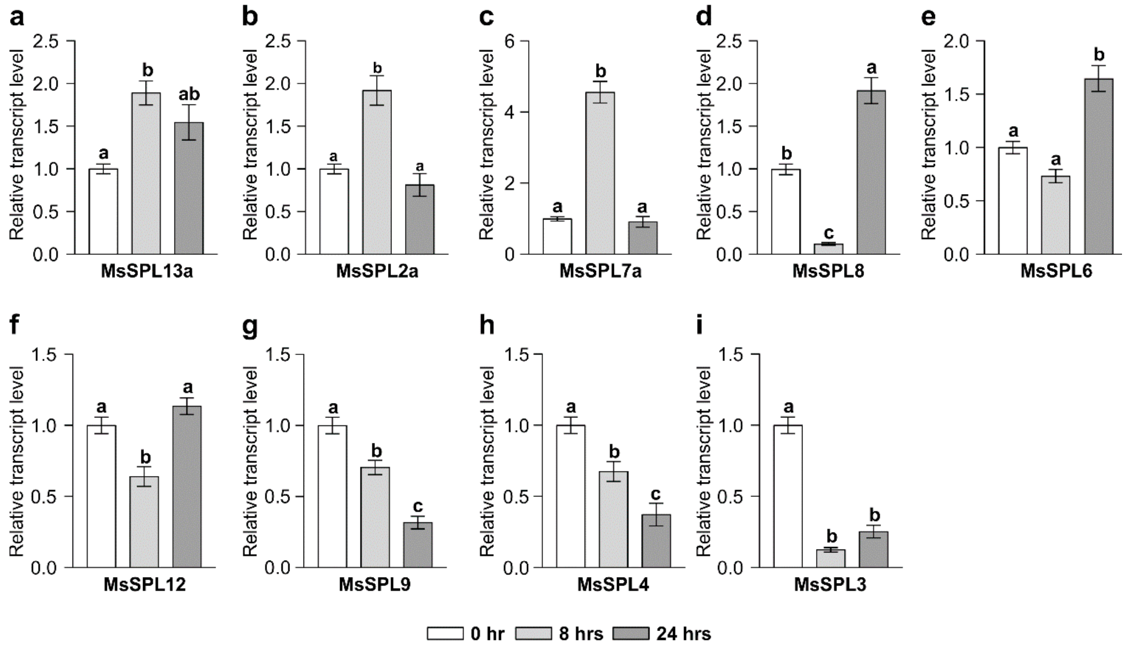

Figure S1. Differential expression *MsSPL* genes in roots of 49-day-old alfalfa plants treated with Al for 0, 8, 24 hrs. Each bar plot represents the mean. Error bars represent (SEM). A one-way ANOVA was conducted with n = 4 individual plants. Significant differences detected from the one-way ANOVA in R (version R-4.2.3) were followed by a post hoc Tukey multiple comparison test. Means with the same letters are not significantly different at $p \leq$ (value).

Table S8. Summary of RNA-seq Read Counts and Mapping Statistics

Sample	Raw reads	Clean reads	% Mapped reads	Gene assignment
Al1WT1	69516131	68276177	91.39	44118544 (15.2%)
AL2WT4	63206914	62069700	93.2	40148923 (15.1%)
AL3WT5	72760424	72405401	92.94	49068448 (16.3%)
AL413051	62747605	61711299	93.01	40902612 (15.6%)
AL513054	68842207	67739685	93.05	45623052 (16.0%)
AL613053	62963220	62017332	92.88	41441515 (15.8%)
AL713061	69833888	68683174	93.3	46430492 (15.9%)
AL813063	59658779	58621617	93.05	38001481 (15.1%)
AL913064	62851021	61682068	92.78	39745632 (15.0%)
C1WT4	65327188	64255127	93.28	41526145 (15.1%)
C2WT5	61780217	60325668	93.24	37589817 (14.1%)

C3WT6	61684264	60612479	92.78	40687559 (15.8%)
C413053	61302682	60323684	92.71	39399745 (15.5%)
C513051	64617994	63424721	92.14	40448134 (14.9%)
C613054	72796087	71587592	92.92	47081689 (15.6%)
C713061	71512642	70383923	93.4	47194857 (15.8%)
C813062	64492954	63224904	92.94	40378851 (14.8%)
C913063	66517413	65310389	92.26	41731786 (15.1%)
**Total**	1,182,411,630	1,162,654,940<br>(98.33%)		761519282
Average	65,689,535	64,591,941.11	92.85	42,306,626.78
Average WTC		61,731,091.33		39,934,507
Average SPL13RNAiC		65,709,202.17		42,705,843.67
Average WT Al		67,583,759.33		44,445,305
Average SPL13RNAi Al		63,409,195.83		42,024,130.67

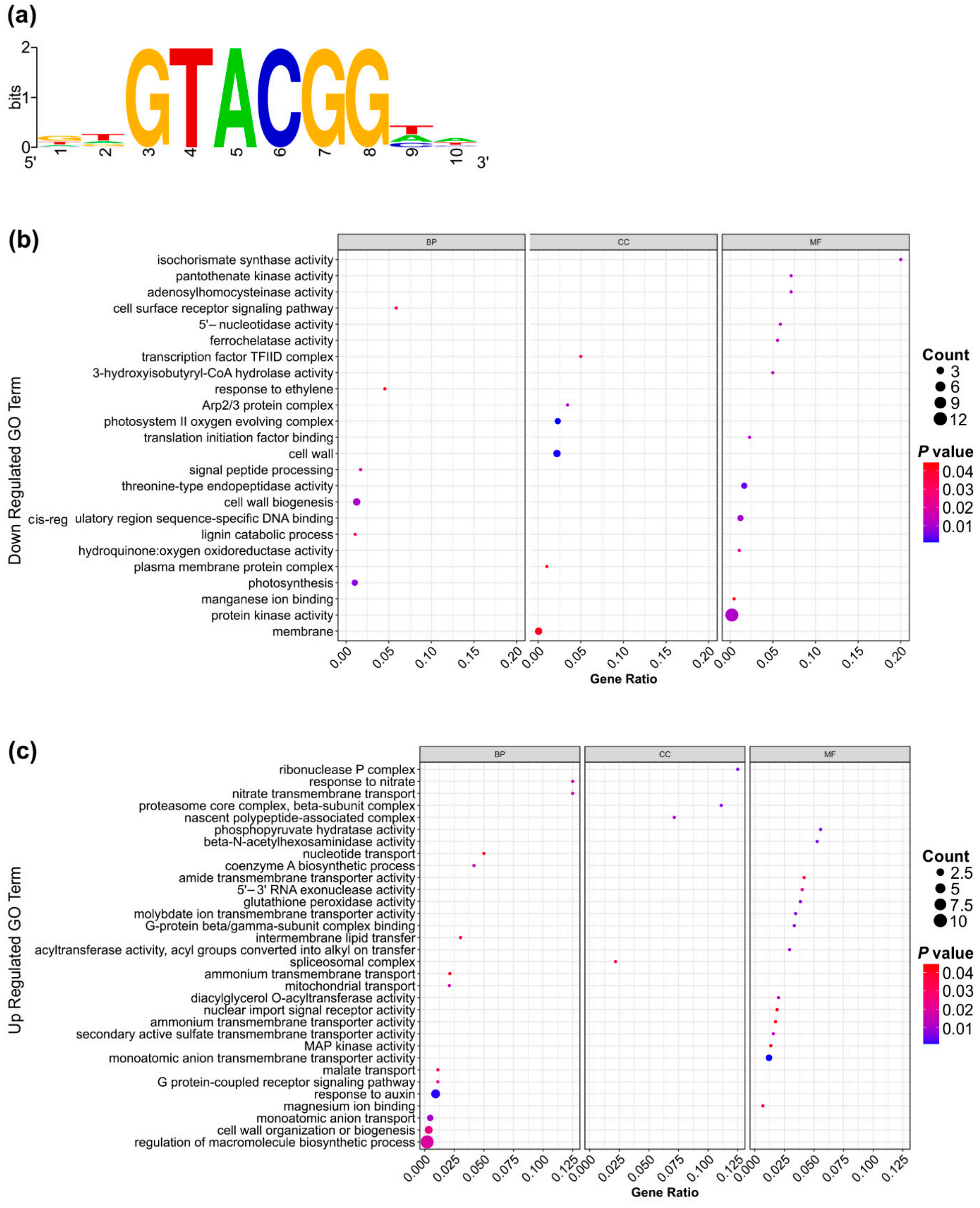

Figure S2. GO term and functional response of differentially expressed genes (DEGs) in SPL13-RNAi compared to WT. Enrichment of the core GTAC motif in the promoter of all DEGs (**a**), Dot plot representing the enriched GO terms

for CC,BP, and MF among the downregulated DEGs (**b**). Dot plot showing the enriched GO terms for CC,BP, and MF among the upregulated DEGs (**c**)

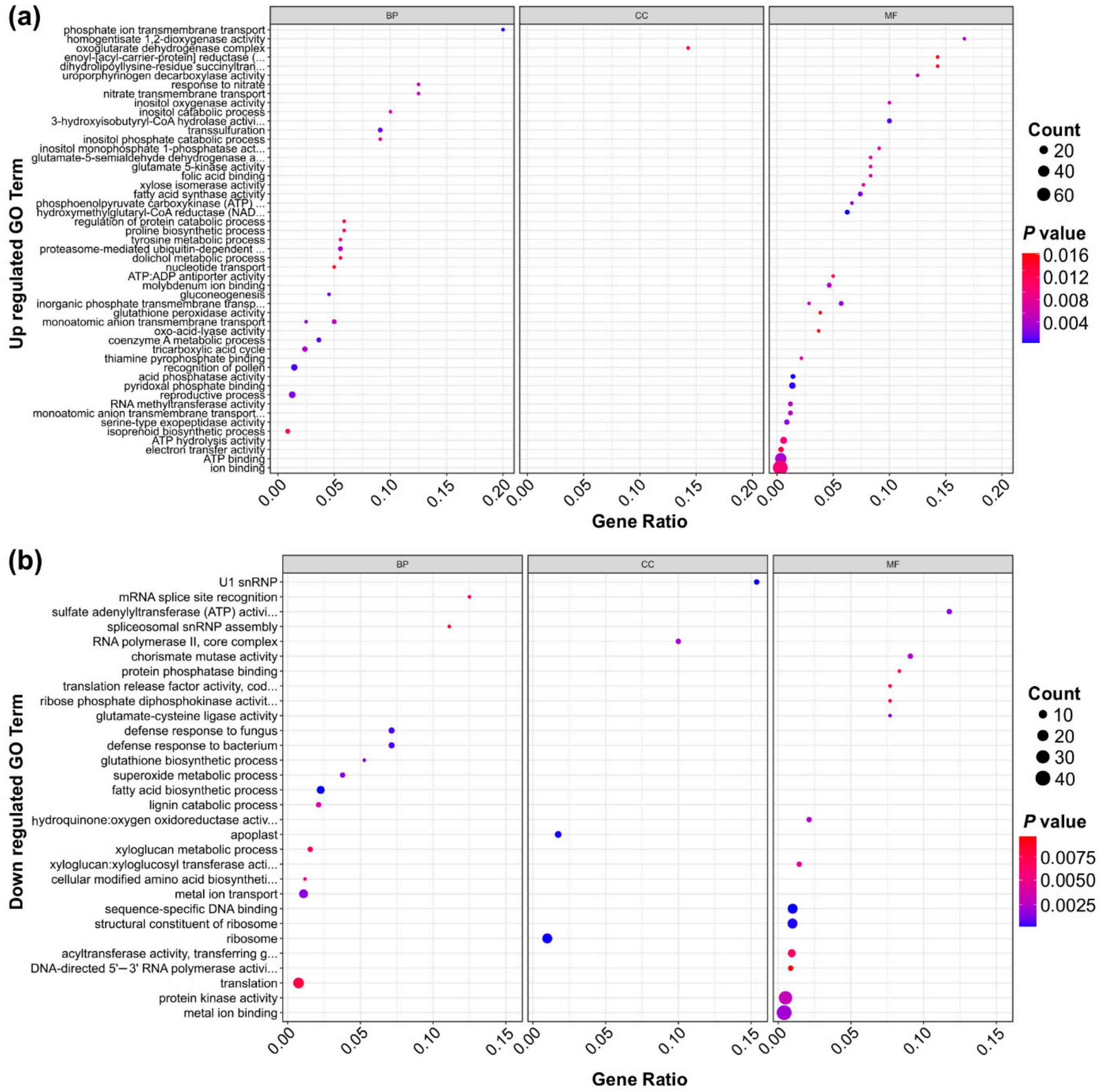

Figure S3. GO-term analysis of genotype-specific comparison of DEGs in WT and SPL13RNAi plants under Al stress and control conditions. Dot plot depicting the enriched GO terms for upregulated DEGs (a). Dot plot depicting the enriched GO term for downregulated DEGs (b).

Tables S9-S18: see Supplementary Sequencing Data
